# NAD+ Metabolism-Related Gene Profile Can Be a Relevant Source of Squamous Cell Carcinoma Biomarkers

**DOI:** 10.3390/cancers16020309

**Published:** 2024-01-11

**Authors:** Ylenia Aura Minafò, Dario Antonini, Elena Dellambra

**Affiliations:** 1 Molecular and Cell Biology Laboratory, Fondazione Luigi Maria Monti, IDI-IRCCS, Via dei Monti di Creta, 104, 00167 Rome, Italy; y.minafo@idi.it; 2 Department of Biology, University of Naples Federico II, 80126 Naples, Italy; dario.antonini@unina.it

**Keywords:** squamous cell carcinoma, NAD+, metabolism, biomarker, TCGA database

## Abstract

**Simple Summary:**

Poor survival rates of squamous cell carcinomas (SCCs) are due in part to a limited number of reliable biomarkers and molecular targets. NAD+ metabolism plays a relevant role in SCC chemoprevention and therapy and is found dysregulated in several tumors. Therefore, the aim of our study was to identify new biomarkers based on NAD+ metabolism-related gene (NMRG) expression. Our findings indicate that NAD+ metabolism-related gene profiles can be an important source of SCC biomarkers and potential therapeutic targets. These findings agree with SCC preclinical and clinical studies using NAD+ precursors. Further in-depth studies on identified NMRGs may provide additional insights into SCC pathogenesis and improve therapeutic choice.

**Abstract:**

Poor survival rates of squamous cell carcinomas (SCCs) are associated with high recurrence, metastasis, and late diagnosis, due in part to a limited number of reliable biomarkers. Thus, the identification of signatures improving the diagnosis of different SCC types is mandatory. Considering the relevant role of NAD+ metabolism in SCC chemoprevention and therapy, the study aimed at identifying new biomarkers based on NAD+ metabolism-related gene (NMRG) expression. Gene expression of 18 NMRGs and clinical-pathological information for patients with head and neck SCC (HNSCC), lung SCC (LuSCC), and cervix SCC (CeSCC) from The Cancer Genome Atlas (TCGA) were analyzed by several bioinformatic tools. We identified a 16-NMRG profile discriminating 3 SCCs from 3 non-correlated tumors. We found several genes for HNSCC, LuSCC, and CeSCC with high diagnostic power. Notably, three NMRGs were SCC-type specific biomarkers. Furthermore, specific signatures displayed high diagnostic power for several clinical-pathological characteristics. Analyzing tumor-infiltrating immune cell profiles and PD-1/PD-L1 levels, we found that NMRG expression was associated with suppressive immune microenvironment mainly in HNSCC. Finally, the evaluation of patient survival identified specific genes for HNSCC, LuSCC, and CeSCC with potential prognostic power. Therefore, our analyses indicate NAD+ metabolism as an important source of SCC biomarkers and potential therapeutic targets.

## 1. Introduction

Squamous cell carcinoma (SCC) is an epithelial tumor that occurs in organs covered with squamous epithelium, such as skin, lips, mouth, esophagus, urinary tract, prostate, lungs, vagina, and cervix [1]. The SCC incidence is different across tissues as risk factors vary in relation to the site-specific protective function of the epithelia [1]. Three of the major SCC types are head and neck SCC (HNSCC), SCC of the lung (LuSCC), and cervix SCC (CeSCC). HNSCC is the sixth most common cancer worldwide and consists of a group of tumors that arise from squamous mucosal surfaces, including nasal and oral cavities, the nasopharynx, the oropharynx, the hypopharynx, and the larynx. Primary risk factors for HNSCC include tobacco and alcohol use, alpha-type human papillomavirus (HPV) infection, age, and genetic predisposition [1,2]. LuSCC is a type of non-small cell lung cancer (NSCLC) and occurs in the central part of the lung or the main airway. The main risk factor for LuSCC is tobacco smoke. Other risk factors include age, family history, exposure to asbestos or air pollution, and genetic factors [1,3]. CeSCC is the most common gynecological tumor and the fourth leading cause of cancer-related death in women. The primary risk factor for CeSCC is persistent infections with alpha-type HPVs [3,4]. 

SCCs are characterized by local invasive patterns, high recurrence, and metastasis. Therefore, the treatment of SCC is comparable across the different anatomical sites [1]. Standard treatments include surgery, chemotherapy, or radiotherapy in early and advanced curable disease, whereas targeted systemic therapies or immunotherapy are used in locally advanced and metastatic tumors [5,6]. However, late diagnosis, recurrence, and metastases are associated with poor survival rates. Despite advances in diagnostic methods and combined treatments, the survival rate has not significantly improved over the last 30 years due in part to a lack of reliable early diagnostic biomarkers and a limited number of molecular targets for patients with advanced disease [1,7,8]. 

SCCs from different body sites exhibit similar histopathological features [3]. SCC development is a multistep process involving the accumulation of multiple genetic alterations modulated by genetic predisposition and environmental insults. SCCs share many phenotypic and molecular characteristics (e.g., mutations in genes involved in squamous cell differentiation and cell adhesion) that distinguish SCC from other cancer types. However, mutations of some genes appear more frequent or potentially specific to a given SCC type [1,7,8]. Thus, the identification of new diagnostic biomarkers or specific signatures that correlate with pathophysiological characteristics can improve therapeutic choice.

Energy metabolism reprogramming is one of the hallmarks of cancer [9]. Nicotinamide adenine dinucleotide (NAD+) and its metabolites play a key role in the control of energy homeostasis, enabling the cells to adapt to environmental changes, including nutrient perturbation, genotoxic factors, infections, inflammation, and xenobiotics. Therefore, NAD+ depletion can be involved in many critical steps of tumorigenesis, such as genome instability, metabolic changes, cell growth, inflammation, and immune response [10,11]. Preclinical studies indicate that the increase of NAD+ levels restores the bioenergetics, redox balance, and signaling pathways, ameliorating the adverse effects of pathophysiological conditions and offering a promising option for therapy [10,11,12]. NAD+ levels could be restored by dietary NAD+ precursors (e.g., nicotinamide (NAM), nicotinamide mononucleotide (NMN) and nicotinamide riboside (NR)), inhibitors of NAD+-consuming enzymes, management of the NAD+ biosynthesis via controlling NAD+-biosynthesis enzymes or improving NAD+ bioavailability through caloric restriction or exercise [11]. Clinical trials indicate that NAM oral administration displays chemopreventive effects against skin SCC development and recurrence in high-risk immunocompetent [13] and in immunocompromised patients [14]. Moreover, NAM improved the efficacy of radiotherapy against HNSCC and laryngeal SCC [15,16]. Trials assessing the efficacy of NAM when prescribed synergistically with targeted therapies in advanced NSCLC patients are ongoing [12].

Given the growing evidence of the relevant roles that NAD+ metabolism plays in cancer initiation and progression, the aim of the present study was to identify molecular profiles based on NAD+ metabolism-related gene (NMRG) expression from The Cancer Genome Atlas (TCGA) showing diagnostic or prognosis power for HNSCCs, LuSCCs, and CeSCCs and, thus, can be considered potential therapeutic targets.

## 2. Materials and Methods

### 2.1. Data Source

Gene expression profiles, clinical characteristics, and survival information for 504 patients with HNSCC, 501 with LuSCC, and 306 with CeSCC, and corresponding normal specimens (44, 49, and 3 samples, respectively) were extracted from The Cancer Genome Atlas (TCGA) database (https://portal.gdc.cancer.gov/, accessed on 15 November 2022). Due to the small number of normal samples in the TCGA CeSCC database, we further integrated TCGA and Genotype-Tissue Expression (GTEx) databases (https://gtexportal.org/home/datasets, accessed on 15 November 2022) to obtain 22 normal samples.

Low or undetectable *ENPP3* expression is observed in the majority of HNSCC tumor samples (93%). Likewise, *NMRK2* expression is low or undetectable in HNSCC, LuSCC, and CeSCC samples. Due to this issue, *ENNP3* and *NMRK2* were excluded from all analyses restricted to tumor samples.

Receiver Operating Characteristic (ROC) curve analysis

ROC curves were generated on GraphPad Prism 9. The area under the curve (AUC) is plotted as sensitivity% (True Positive Rate) vs. 100%—specificity% (False Positive Rate).

### 2.2. Principal Component Analysis (PCA)

The PCA analysis was performed for 504 HNSCC, 501 LuSCC, 306 CeSCC, 537 Kidney Renal clear cell Carcinoma (KIRC), 168 Glioblastoma (GBM), and 263 Sarcoma (SARC) TCGA patients. TCGA expression (log2 of TPM) of 16 selected genes of nicotinamide metabolism or of glycolysis was used to calculate the principal components.

### 2.3. Immune Microenvironment Analysis

Correlation of NMRG expression with immune infiltration level in HNSCCs, LuSCCs, and CeSCCs (TCGA) was performed using the Tumor IMmune Estimation Resource (TIMER) web application (https://cistrome.shinyapps.io/timer/, accessed on 15 November 2022) [17,18]. The purity-corrected partial Spearman’s rho value and statistical significance were calculated.

### 2.4. Correlation with PD-1 and PD-L1

The correlation coefficient between PD-1 or PD-L1 and genes involved in nicotinamide metabolism was calculated by Spearman’s Rho test using TIMER2.0 (http://timer.cistrome.org/, accessed on 15 November 2022) [19]. Purity adjustment of TCGA HNSCC, LuSCC, and CeSCC was performed before the Spearman calculation.

### 2.5. Survival Analysis

Mantel-Cox analysis was performed between NMRGs with high or low expression and overall survival (OS) in 504 HNSCC, 501 LuSCC, and 306 CeSCC patients. High or low expression was defined as relating to the mean of the log2(TPM) in the tumor samples of each tumor type.

### 2.6. Statistical Analysis

All data processing steps and statistical analyses were performed using GraphPad Prism 9.

Statistical significance of differential gene expression levels in tumor samples compared to control specimens and in patients’ stratification was calculated by a two-tailed Mann-Whitney test. Multiple logistic regression was performed to generate multiple ROC curves on combined expression values in patients’ stratification. A Pearson correlation index and statistical significance were calculated to perform a correlation analysis of NMRGs involved in patients’ survival with immune checkpoint expression.

## 3. Results

### 3.1. NMRGs and Their Ability as SCC-Type Discriminating Biomarkers

NAD+ is an essential metabolite for cellular homeostasis and its metabolism is characterized by a balance of synthesis, consumption, and regeneration. NAD+ acts as a co-enzyme for multiple redox reactions as well as a substrate for NAD+-consuming enzymes (i.e., sirtuins, PARPs, and cADPRSs such as CD38) that coordinate genomic stability, epigenetic status, and inflammation [10].

**Figure 1:**
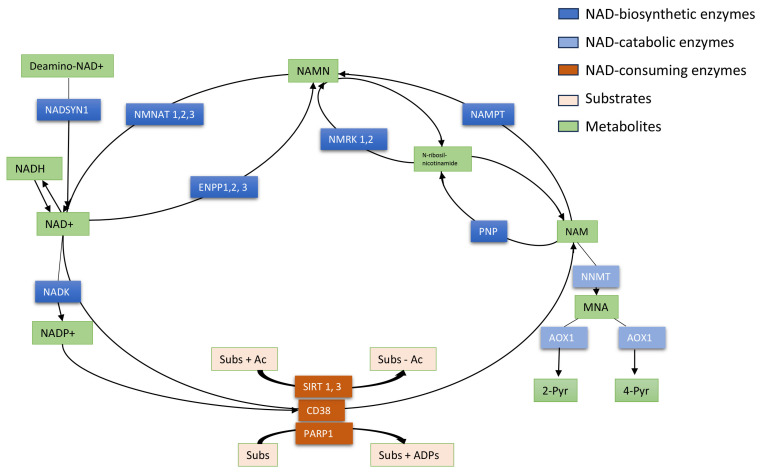
Schematic representation of NAD+ metabolism. NAD+ is an essential coenzyme of redox reactions for the production of adenosine triphosphate (ATP) and is involved in several metabolic processes. Nicotinamide (NAM) is converted to NAD+ via the salvage pathway, which represents the major pathway of NAD+ biosynthesis in mammals. NAM phosphoribosyltransferase (NAMPT) is the “rate-limiting” enzyme that catalyzes the first step in the biosynthesis of NAM mononucleotide (NAMN) from NAM. NMN adenyltransferases (NMNATs) utilize ATP to generate NAD+, which can also be directly converted into NADP+ by NAD kinase (NADK). NAMN can also be synthesized by NMRKs from N-ribosil-nicotinamide which is metabolized by PNP from NAM. In a consuming way, the cyclic ADP-ribose synthase (cADPRS) CD38 hydrolyzes NAD+ to NAM. Poly-ADP-ribose-polymerases (PARPs) use NAD+ as a co-substrate to “ADP-ribosylate” target proteins (Sub), generating NAM. Sirtuins (SIRT) depend on NAD+ to deacetylate specific substrates (Sub) generating NAM. At least, in the catabolic way NAM is methylated to 1-methyl-NAM (MNA) by NAM-N-methyltransferase (NNMT) and then oxidized to l-methyl-2-pyridone-5-carboxamide (2-Pyr) and l-methyl-4-pyridone- 5-carboxamide (4-Pyr).

We investigated the gene expression of the foremost NMRGs (Figure 1) in specimens of HNSCC, LuSCC, and CeSCC using the TCGA dataset to assay their ability to discriminate cancer samples from normal controls. Namely, *AOX1*, *NNMT*, *NAMPT*, *NMNAT1*, *NMNAT2*, *NMNAT3*, *ENPP1*, *ENPP2*, *ENPP3*, *NMRK1*, *NMRK2*, *PNP*, *NADK*, *NADSYN1*, *SIRT1*, *SIRT3*, *CD38,* and *PARP1* were investigated in each SCC type.

Numerous cancer samples and corresponding controls for each SCC type are reported in Table S1 which describes the clinicopathologic characteristics of analyzed patients. The distribution of clinicopathologic parameters in the paired cohorts was comparable. Due to the small number of normal CeSCC samples in the TCGA database, TCGA and GTEx data were integrated for further analysis.

**Figure 2:**
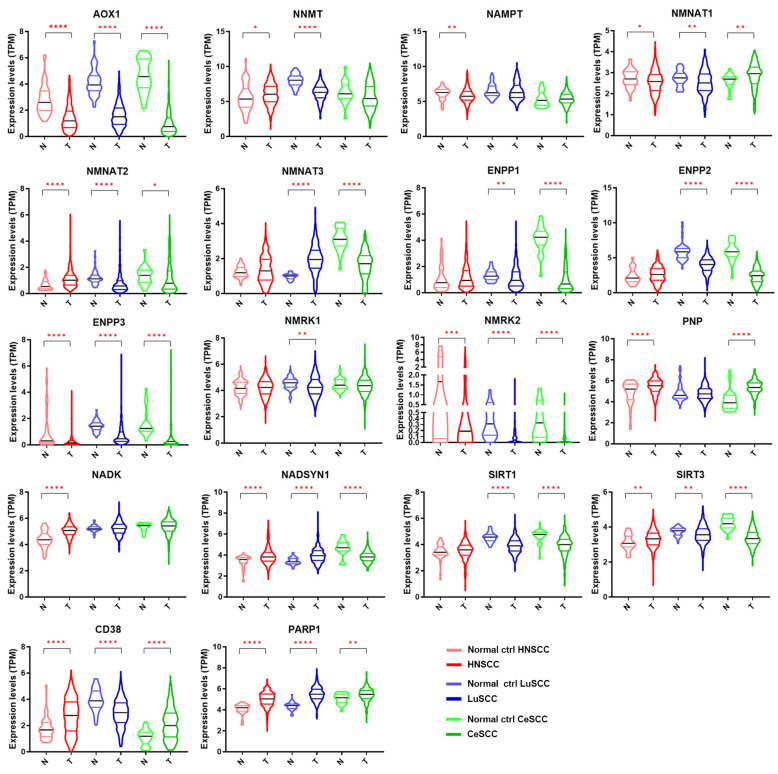
Gene expression levels of 18 NMRGs in HNSCC, LuSCC, and CeSCC compared to corresponding controls. Violin plots representing the gene expression data of *AOX1*, *NNMT*, *NAMPT*, *NMNAT1*, *NMNAT2*, *NMNAT3*, *ENPP1*, *ENPP2*, *ENPP3*, *NMRK1*, *NMRK2*, *PNP*, *NADK*, *NADSYN1*, *SIRT1*, *SIRT3*, *CD38,* and *PARP1* from the TCGA-HNSCC, TCGA-LuSCC, and TCGA-CeSCC plus endo- and ectocervix from GTEx datasets. Values on the *y*-axis represent the log2 of the normalized mRNA sequencing counts (TPM) for each NMR gene. On the *x*-axis, normal (N) and primary tumors (T) are indicated. Different colors represent tumor types and normal tissues. *p* Values were calculated using a two-tailed Mann-Whitney test. * *p* ≤ 0.05, ** *p* ≤ 0.01, *** *p* ≤ 0.001, **** *p* ≤ 0.0001. HNSCC: head and neck squamous cell carcinoma; LuSCC: lung squamous cell carcinoma; CeSCC: cervix squamous cell carcinoma; N: control specimens; T: tumor samples.

The expression levels of each NMRG in 504 patients with HNSCC, 501 with LuSCC, and 306 with CeSCC compared to corresponding normal specimens (44, 49, and 22 samples, respectively) are shown in Figure 2. The modulation of different NMRGs based on SCC types is reported in Figure S2. Different expression patterns between patient tumor specimens and corresponding normal controls were observed for most genes. Specifically, some genes were significantly modulated in all three tumors in the same way: *AOX1*, *ENPP3*, and *NMRK2* were down-modulated in tumor specimens, whereas *PARP1* was up-regulated. On the contrary, some genes were specifically modulated in a single tumor type. In fact, HNSCC was characterized by a significant decrease of *NAMPT* and an increase of *NADK* whereas LuSCC was characterized by a decrease of *NMRK1.* Although *ENPP1* was down-modulated in two tumor types, the differential expression was more significant in CeSCC.

Other genes were modulated in a different way in all three SCCs (*NMNAT1*, *NMNAT2*, *NADSYN1*, *SIRT3,* and *CD38*) or in two tumor types (*NNMT*, *NMNAT3*, *ENPP2*, *PNP*, and *SIRT1*). Although *NNMT1* was modulated in two tumor types, the differential expression was highly significant in LuSCC.

Receiver operating characteristic (ROC) analysis is used in clinical epidemiology to quantify how accurately diagnostic assays can discriminate between two patient states (e.g., “diseased” and “nondiseased”). Specifically, the area under the curve (AUC) has a meaningful interpretation for discrimination. AUC values range from 0.5 to 1, with 1 indicating 100% ability to discriminate “diseased” from “undiseased” cases.

**Figure 3:**
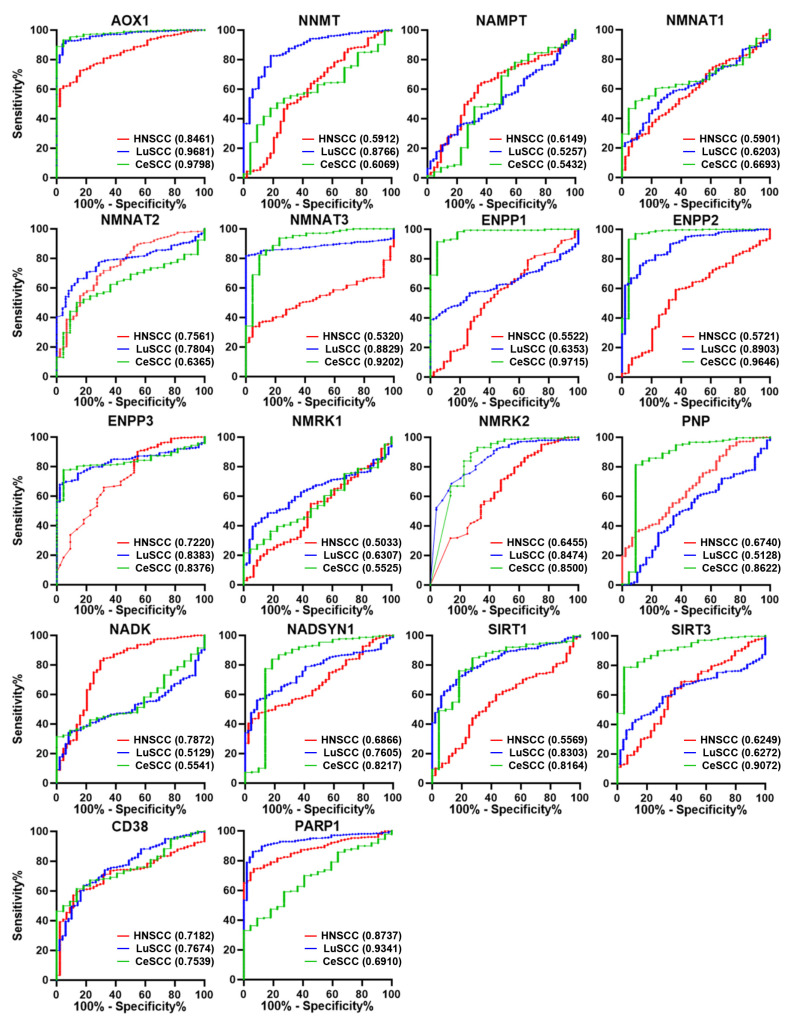
ROC analysis on the expression values of the 18 NMRGs. Overlapped ROC curves based on *AOX1*, *NNMT*, *NAMPT*, *NMNAT1*, *NMNAT2*, *NMNAT3*, *ENPP1*, *ENPP2*, *ENPP3*, *NMRK1*, *NMRK2*, *PNP*, *NADK*, *NADSYN1*, *SIRT1*, *SIRT3*, *CD38*, and *PARP1* gene expression values for the three SCC types are represented in the same plot for each gene. Red lines indicate HNSCC; blue lines indicate LuSCC; green lines indicate CeSCC. The area under the curve (AUC) is plotted as sensitivity% vs. 100%—specificity%. AUC and *p* values are reported for each gene highlighted with the tumor’s corresponding color. HNSCC: head and neck squamous cell carcinoma; LuSCC: lung squamous cell carcinoma; CeSCC: cervix squamous cell carcinoma.

To investigate the diagnostic power of NMRGs in discriminating each SCC type from corresponding controls, the AUC was calculated for each gene and reported in Tables S2–S4. The AUC comparison based on each NMRG and on the SCC type is shown in Figure 3 and Figure S3, respectively.

Considering genes with AUC > 0.7 and *p* ≤ 0.0001, we found 6 genes for HNSCC (*AOX1*, *NMNAT2*, *ENPP3*, *NADK*, *CD38*, and *PARP1*), 11 genes for LuSCC (*AOX1*, *NNMT*, *NMNAT2*, *NMNAT3*, *ENPP2*, *ENPP3*, *NMRK2*, *NADSYN1*, *SIRT1*, *CD38,* and *PARP1*), and 11 genes for CeSCC (*AOX1*, *NMNAT3*, *ENPP1*, *ENPP2*, *ENPP3*, *NMRK2*, *PNP*, *NADSYN1*, *SIRT1*, *SIRT3*, and *CD38*) with a good ability to differentiate each SCC from control samples (Tables S2–S4 and Figure S3).

Interestingly, some discriminating genes were typical of a SCC type (Figure 3). *NADK* was able to discriminate HNSCC from healthy controls (AUC = 0.7872; *p* < 0.0001) but not LuSCC (AUC = 0.5129; *p* = 0.7660) or CeSCC (AUC = 0.5541; *p* = 0.3962). Likewise, *NNMT* (AUC = 0.8766; *p* < 0.0001) discriminated LuSCC from controls but not HNSCC (AUC = 0.5912; *p* < 0.0448) or CeSCC (AUC = 0.6069; *p* = 0.0940). Furthermore, CeSCC was well discriminated by *ENPP1* (AUC = 0.9715; *p* < 0.0001), *PNP* (AUC = 0.8622; *p* < 0.0001), and *SIRT3* (AUC = 0.9072; *p* < 0.0001), different from HNSCC (*ENPP1*:AUC = 0.5522; *p* = 0.2505; *PNP*:AUC = 0.6740; *p* = 0.001; *SIRT3*:AUC = 0.6249; *p* = 0.006) and LuSCC (*ENPP1*:AUC = 0.6353; *p* = 0.0018; *PNP*:AUC = 0.5128; *p* = 0.7671; *SIRT3:* AUC = 0.6272; *p* = 0.0033).

Considering both differential expression and ROC analysis, three differentially expressed NMRGs are able to discriminate tumors from control samples selectively in each SCC type. Thus, a significant increase of *NADK*, a decrease of *NNMT1*, and a decrease of *ENPP1* can be considered relevant specific biomarkers for HNSCC, LuSCC, or CeSCC, respectively.

### 3.2. NMRGs and Their Ability as SCC Discriminating Biomarkers

As SCCs share histological features and many molecular characteristics [8], we investigated whether the NMRG profile was typical of SCC or was common to several tumors.

**Figure 4:**
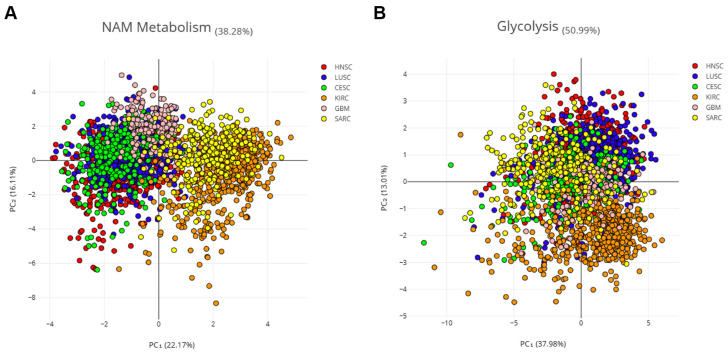
PCA on three SCC and three non-correlated tumors. Principal component analysis (PCA) of six cancer types, three SCCs (HNSCC, LUSCC, and CESCC), and three non-correlated tumors (KIRC, SARC, and GBM). Log2 of the normalized mRNA sequencing counts (TPM) of 16 NMRGs (**A**) or 16 glycolysis-related genes (**B**) was used to perform the PCA. *ENPP3* and *NMRK2* were excluded from this analysis which considers only the expression in tumor samples, since low or undetectable *ENPP3* expression is observed in the majority of HNSCC tumor samples and low or undetectable *NMRK2* expression is observed in the majority of HNSCC; LuSCC and CeSCC tumor samples.

This hypothesis was verified using principal component analysis (PCA) with the combined expression data of 16 NMRGs (*AOX1*, *NNMT*, *NAMPT*, *NMNAT1*, *NMNAT2*, *NMNAT3*, *ENPP1*, *ENPP2*, *NMRK1*, *PNP*, *NADK*, *SIRT1*, *SIRT3*, *CD38,* and *PARP1)* on samples from three SCC and three non-correlated tumors. Specifically, we considered kidney renal clear cell carcinoma (KIRC), which is a non-squamous epithelial tumor, sarcoma (SARC), and glioblastoma (GBM) (Figure 4A). The NMRG profile allowed a clear separation of SCCs from other tumors, mainly KIRK and SARC, indicating that such a profile seems specific for SCC.

Aerobic glycolysis is persistently activated in tumor cells and several cancers are characterized by glycolysis-related gene signature [20]. Thus, 16 genes from the glycolysis pathway (i.e., *PKM*, *TPI1*, *PFKP*, *PGAM1*, *PFKM*, *FOXK1*, *ENO1*, *ALDOA*, *ENO2*, *ENO3*, *FOXK2*, *GPI*, *HK1*, *PFKL*, *PGK1,* and *GAPDH*) were used as the control (Figure 4B). PCA analysis showed that the glycolysis-related profile is not able to cluster patients with different tumors, indicating that such a profile seems common for all six tumors.

Therefore, the NMRG profile can be considered a relevant biomarker of SCC which is able to discriminate these tumors from KIRC, SARC, and GBM.

### 3.3. NMRG Expression and Risk Factors

Beyond site-specific risk factors, age, and gender are common determinants for SCC tumors [1].

In Figure S1, NMRGs with significant differential expression in age and gender groups of SCC patients are reported. *NNMT1* was significantly up-regulated in HNSCC patients aged 81–100 years old compared to young patients (21–40 years), whereas *NADSYN1* was more expressed in patients aged 41–60 years old. In keeping with the literature data, *CD38* expression significantly increased with the age of HNSCC patients and probably was related to reported tissue NAD+ depletion [21,22]. *AOX1* and *ENPP2* displayed a significant differential expression between LuSCC patients aged 41–60 years old and 61–80 years old. An age-related increased expression was observed for *ENPP3* in LuSCC patients. In CeSCC, an age-related decreased and increased expression was found for *NNMT1* and *NMNAT3*, respectively. *NMNAT1* significantly increased only in the 61–80-year-old patient group, whereas *NMNAT2* significantly decreased in the 81–100-year-old group.

Males with HNSCC displayed a significantly increased expression of *NMNAT3* and *PARP1* with respect to females, whereas males with LuSCCs showed increased levels of *NAMPT*.

Thus, only a few NMRGs can be considered age- or gender-related in those SCC samples.

### 3.4. NMRGs as Diagnostic Biomarkers for Patient Stratification

SCCs are classified according to histological features (e.g., stage, grade), anatomical site, and, in some types, HPV status (Table S1). Staging and grading of SCC are established prerequisites for management as they influence risk stratification and treatment planning. The TNM system is based on an assessment of the size of the primary tumor (T), involvement of locoregional lymph nodes (N), and metastasis (M). HNSCC staging displays values from 1 to 4, which represent the most advanced forms. 

**Figure 5:**
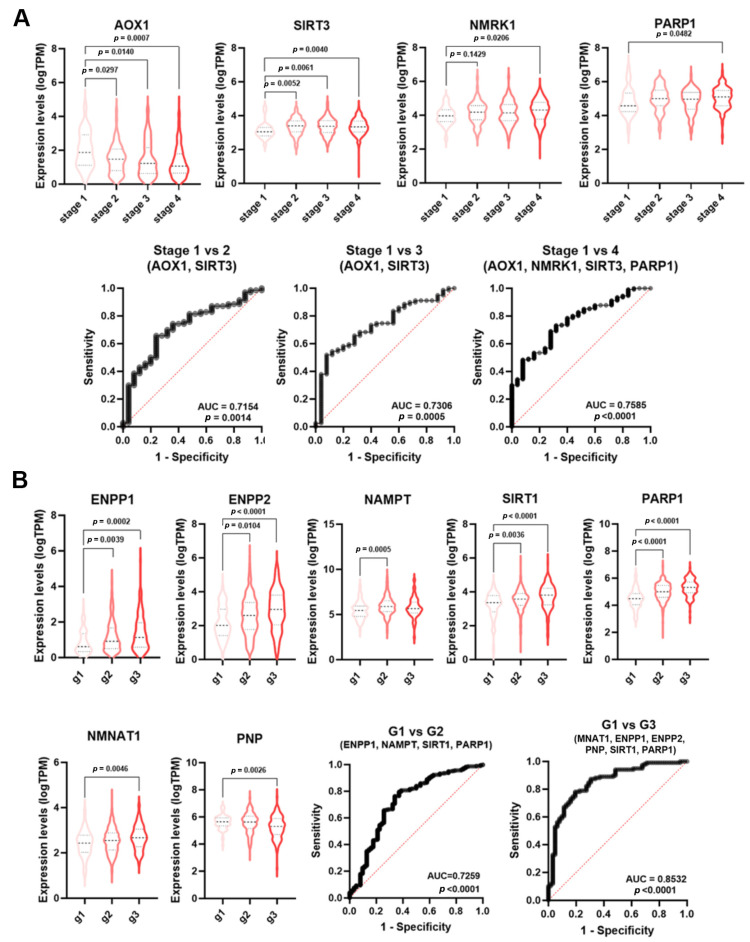
NMRGs as diagnostic biomarkers for HNSCC staging and grading. Differential gene expression levels of selected genes (violin plots) and ROC curve on their combined expression values for HNSCC patients staging (**A**) and grading (**B**). HNSCC: head and neck squamous cell carcinoma.

As reported by ROC analysis, some single genes displayed a low diagnostic power (AUC < 0.7) for HNSCC staging (Table S2). However, NMRGs with significant AUC values (*AOX1*, *SIRT3*, *NMRK1*, *PARP1*) were differentially expressed among tumor stages (Figure 5A). Thus, multiple logistic analysis and ROC analysis were carried out on the combined expression profile of these NMRGs with significant AUC values to identify a diagnostic signature.

As shown in Figure 5A, the signature combining the expression values of *AOX1* and *SIRT3* was able to discriminate stage 1 from stage 2 HNSCC samples (AUC = 0.7154, *p* = 0.0014) and stage 1 from stage 3 (AUC = 0.730, *p* = 0.0005). The signatures *AOX1*, *NMRK1*, *SIRT3*, and *PARP1* were able to discriminate stage 1 from stage 4 HNSCC (AUC = 0.7585; *p* < 0.0001). No single NMRG or specific signature was able to discriminate LuSCC stages (Table S3).

Histopathological grading is based on the differences in tumor differentiation. Low-grade or grade 1 HNSCC are well-differentiated tumors, whereas high-grade or grade 3 HNSCC are poor or undifferentiated lesions. Likewise staging, single genes displayed a low diagnostic power for HNSCC grading (Table S2). However, NMRGs with significant AUC values (*ENPP1*, *ENPP2*, *NAMPT*, *SIRT1*, *PARP1*, *NMNAT1*, and *PNP*) were differentially expressed among tumor grades (Figure 5B). Multiple logistic analysis and ROC analysis on the combined expression profile of these genes with significant AUC value (Table S2) identified a diagnostic signature (*NAMPT*, *ENPP1*, *SIRT1*, and *PARP1*) able to discriminate grade 1 from grade 2 HNSCC (AUC = 0.7259; *p* < 0.0001). Furthermore, the signature *NMNAT1*, *ENPP1*, *ENPP2*, *PNP*, *SIRT1*, and *PARP1* were able to discriminate grade 1 from grade 3 HNSCC (AUC = 0.8532; *p* < 0.0001). No single NMRG or specific signature was able to discriminate CeSCC grade (Table S4).

**Figure 6:**
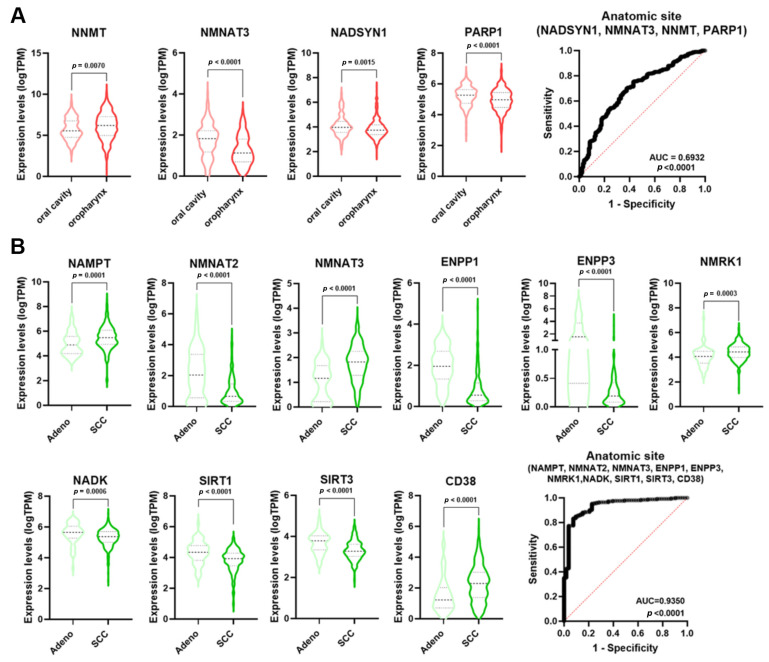
NMRGs as diagnostic biomarkers for HNSCC and CeSCC anatomic site. Differential gene expression levels of selected genes (violin plots) and ROC curves on their combined expression values for HNSCC (**A**) and CeSCC (**B**) anatomic site. HNSCC stratification violin plots are colored in different shades of red; CeSCC stratification violin plots are colored in different shades of green. HNSCC: head and neck squamous cell carcinoma; CeSCC: cervix squamous cell carcinoma.

The incidence of HNSCC greatly varies depending upon the anatomic region [23]. Single NMRGs displayed a low diagnostic power for discriminating the oropharynx specimens from those of the oral cavity (Table S2). However, NMRGs with significant AUC values (*NNMT*, *NMNAT3*, *NADSYN1*, and *PARP1*) were differentially expressed between these anatomic regions (Figure 6A). Notably, multiple logistic analysis and ROC analysis on the combined expression profile of these genes indicated that the signature had a high diagnostic power for discriminating the oropharynx from oral cavity HNSCCs (AUC = 0.6932; *p* < 0.0001).

Considering the histological type of CeSCC, single NMRGs displayed a low diagnostic power for discriminating the adenocarcinoma from SCC (Table S4). However, NMRGs with significant AUC values (*NAMPT*, *NMNAT2*, *NMNAT3*, *ENPP1*, *ENPP3*, *NMRK1*, *NADK*, *SIRT1*, *SIRT3,* and *CD38*) were differentially expressed between histological types (Figure 6B). Multiple logistic analysis and ROC analysis on the combined expression profile of these genes identified that signature as a discriminant for adenocarcinoma from SCC of the cervix (AUC = 0.935; *p* < 0.0001).

**Figure 7:**
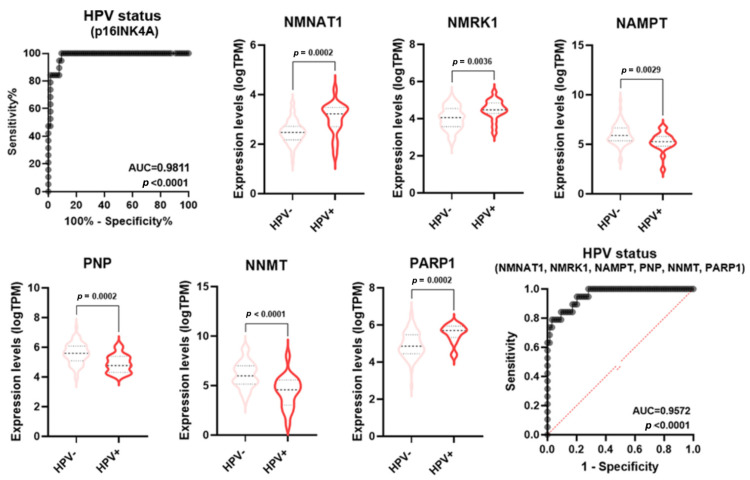
NMRGs as diagnostic biomarkers for HPV infection status. Differential gene expression levels of selected genes (violin plots) and ROC curve on their combined expression values for HPV infection status in HNSCC specimens. HNSCC: head and neck squamous cell carcinoma.

Numerous pieces of evidence indicate molecular and clinical differences in mutations, gene expression regulation, treatment responses, and patient survival rates between HPV-negative and HPV-positive HNSCC [23]. Specifically, HPV-positive tumors usually display a better prognosis than HPV-negative tumors. p16 (INK4A) staining is a widely used and generally accepted surrogate tool to identify HPV association and, therefore, to classify HNSCC as either associated or not associated with the viral infection [23]. In keeping with the literature data, the ROC analysis of *p16* expression in HPV positive vs negative samples displayed an AUC = 0.9811 (*p* < 0.0001) (Figure 7). Some single NMRGs (*NNMT*, *NAMPT*, *NMNAT1*, *NMRK1*, *PNP,* and *PARP1*) displayed a good diagnostic power for discriminating HPV status of HNSCC (Table S2) and were differentially expressed between HPV-negative and HPV-positive groups (Figure 7). Furthermore, the signature combining these NMRGs displayed a very high diagnostic power (AUC = 0.9572; *p* < 0.0001) similar to what was measured with the p16 expression (Figure 7).

Therefore, specific NMRG signatures display high diagnostic power for several clinical-pathological characteristics and their validation may be helpful for diagnosis and personalized treatments.

### 3.5. NMRGs and Immune Microenvironment Characterization

Current staging and grading systems are centered on the tumor per se rather than stroma and host responses. Thus, they are informative for prognosis but not exhaustive, as the immune microenvironment highly influences SCC development and the response to immunotherapy [24,25]. Comprehensive characterization of tumor-infiltrating immune (TII) cells in solid tumors has been increasingly recognized as a novel and robust prognostic factor. Notably, NAD+ metabolism may regulate the function of innate and adaptive immune cells, which can act as tumor suppressors or promoters, and, therefore, contribute to inflammatory response [26]. Therefore, the characterization of the SCC immune microenvironment can help the treatment stratification, especially for the new immunotherapeutic drugs.

The amount of six TII cell types (B cells, CD4 T cells, CD8 T cells, neutrophils, macrophages, and dendritic cells) in the tumor microenvironment of HNSCC, LuSCC, and CeSCC was estimated using Tumor IMmune Estimation Resource (TIMER), a web resource for the evaluation of clinical relevance of tumor-immune infiltration. The correlation of NMRG expression with immune infiltration level was further evaluated (Table 1). The data revealed that although TII cells examined were found in all SCC types, the best correlations with NMRG expression were observed in HNSCC. On the contrary, poor correlations were observed in CeSCC.

Considering HNSCC, CD4+ T cells were the most relevant immune cells positively correlated with NMRG expression (13/16 genes). The expression of seven NMRGs (i.e., *AOX1*, *ENPP1*, *ENPP2*, *NADK*, *SIRT1*, *CD38,* and *PARP1*) was positively correlated to infiltrating levels of all TII cells. On the contrary, *PNP* and *SIRT3* expression displayed significantly negative correlations with TII amount.

Considering LuSCC, the levels of neutrophils, macrophages, and CD8+ T cells were significantly positively associated with most of the NMRGs (10/17, 10/17, and 9/17 genes, respectively). The expression of seven NMRGs (i.e., *AOX1*, *NNMT*, *ENPP1*, *ENPP2*, *ENPP3*, *SIRT1,* and *CD38*) positively correlated with an increase of all TII cells.

Considering CeSCC, the levels of B cells, CD4+ T cells, and macrophages were significantly positively associated with most of the NMRGs (7/17, 7/17, and 5/17, respectively). Only *ENPP2* had a significantly positive correlation with the increase of all TII cells.

Our results suggest a potential role of NMRGs in the tumor immune microenvironment changes mainly in HNSCC.

### 3.6. NMRGs and Immune Checkpoints

Immunotherapy treatments are based on the activation or inhibition of molecules that orchestrate the host immune response and display impressive results in different cancer types, increasing survival with less severe side effects. The most important inhibitory targets are CTL4, PD-1 (PDCD1), and its ligand PD-L1 (CD-274) [27]. Notably, NAD+ metabolism drives IFNγ-induced PD-L1 expression to lead to tumor immune evasion [28].

The correlation between NMRGs and PD-1 or PD-L1 expression in HNSCC, LuSCC, and CeSCC was evaluated using TIMER2.0. The Spearman’s Rho correlation values are reported in Table 1.

Similar to what was observed for TII cells, the best correlations of NMRGs with PD-1 or PD-L1 expression were mainly observed in HNSCC. On the contrary, poor correlations were observed in CeSCC.

Our results indicate that NMRG is associated with a suppressive immune microenvironment mainly in HNSCC.

### 3.7. NMRGs as Prognostic Biomarkers

Several factors can affect patients’ prognosis, including a weakened immune system, tumor location, dimension and depth, and recurrence due to therapy resistance. Early diagnosis improves the survival of patients with SCCs. However, most patients cannot be screened early due to the lack of available biomarkers [1].

To investigate if NAD-metabolism genes may be prognostic markers or therapeutic targets, we analyzed how their expression relates to patient survival. Survival data are reported in Table S5. Expression levels were categorized as “high-level” (i.e., above the median value) and “low-level” (i.e., below the median value).

**Figure 8:**
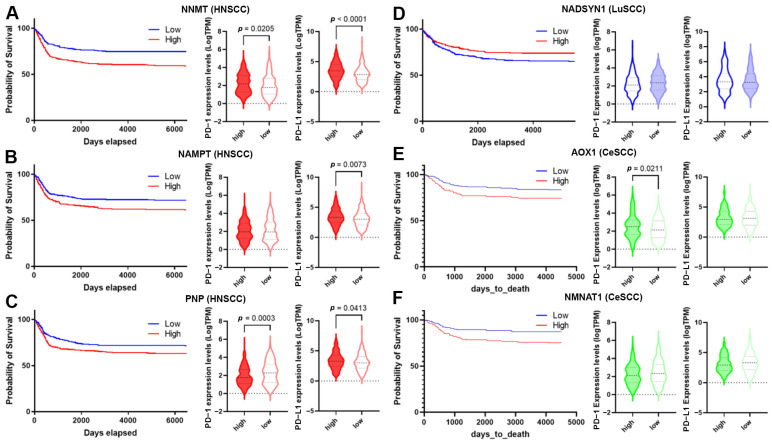
Correlation of NMRG expression with immune infiltrates and checkpoints. (**A**–**F**) The survival curve is reported for each selected gene (blue lines indicate the probability of survival associated with low gene expression, whereas red lines indicate the probability of survival associated with high gene expression) in HNSCC (**A**–**C**); LuSCC (**D**) and CeSCC (**E**,**F**). Violin plots show differential immune checkpoint expression values between high- and low-risk groups for each gene. High-risk groups are represented by filled violins; low-risk groups are represented by empty violins. HNSCC: head and neck squamous cell carcinoma; LuSCC: lung squamous cell carcinoma; CeSCC: cervix squamous cell carcinoma.

High-level expression of *NNMT*, *NAMPT*, and *PNP* was significantly associated with HNSCC’s poor prognosis (Table S5 and Figure 8A–C). Moreover, the down-regulation of *NADSYN1* and up-regulation of *AOX1* and *NMNAT1* were associated with LuSCC and CeSCC poor prognosis, respectively (Table S5 and Figure 8D–F).

The overall survival for HNSCCs and CeSCCs was also investigated using a combination of these specific NMRGs. Notably, the signature “*NNMT*, *NAMPT*, *PNP*” displayed an increased prognostic significance for HNSCCs with respect to single NMRGs (Figure S4). Likewise, the signature “*AOX1*, *NMNAT1*” had a higher prognostic power for CeSCC compared to that of single NMRGs (Figure S4).

To evaluate whether SCC’s poor prognosis was correlated to immune checkpoint alteration, we investigated the expression of *PD-1* and *PD-L1* biomarkers in high- and low-risk groups (Figure 8) and their correlation with NMRG levels (Table 2). *PD-1* and *PD-L1* were significantly up-regulated in high-risk HNSCC with high *NNMT1* levels. In the low-risk group, *NNMT1* levels positively correlated with PD-L1 expression (Figure 8A and Table 2). Considering high-risk HNSCC with high *NAMPT*, *PD-L1* was significantly up-regulated. In the low-risk group, its expression significantly correlated with *NAMPT* levels. *PD-1* expression did not vary between risk groups although in the high-risk group, their levels inversely correlated with *NAMPT* expression. In the low-risk group, *NAMPT* levels positively correlated with *PD-L1* expression (Figure 8B and Table 2). Considering high-risk HNSCC with high *PNP*, *PD-1*, and *PD-L1* were significantly down- and up-regulated, respectively. In the low-risk group, *PD-1* and *PD-L1* levels correlated with *PNP* expression in a negative or positive manner, respectively (Figure 8C and Table 2). *PD-1* and *PD-L1* did not vary between LuSCC risk groups. In the high-risk group, *NADSYN1* levels positively correlated with *PD-L1* expression (Figure 8D and Table 2). Considering high-risk CeSCC with high *AOX1*, *PD-1* was significantly up-regulated. No correlations were found between *AOX1* levels and *PD-1* or *PD-L1* expression (Figure 8E and Table 2). *PD-1* and *PD-L1* did not vary between CeSCC risk groups expressing *NMNAT1*. Furthermore, no correlations were found between *NMNAT1* levels and *PD-1* or *PD-L1* expression (Figure 8F and Table 2).

## 4. Discussion

Given the broad range of tissues in which SCC arises, it represents the most common cancer worldwide [1]. SCCs share several features, including genetic and epigenetic alterations, and the impact of the microenvironment on tumor development and progression.

Several studies showed NAD+ metabolism dysregulation in tumors [10]. However, no NMRGs have been reported as diagnostic or prognostic biomarkers/signatures for HNSCC, LuSCC, and CeSCC.

Using the TCGA dataset, we identified an NMRG profile, composed of 16 genes, able to discriminate these SCC tumors from KIRC, SARC, and GBM specimens. Otherwise, a profile composed of 16 genes encoding for enzymes of the glycolysis did not display a discriminating power between different tumors. Therefore, the NMRG profile can be considered a relevant biomarker of SCCs underlying the significance of NAD+ metabolism in their pathogenesis. This finding agrees with preclinical and clinical studies that evidence a key role of NAD+ precursors in SCC prevention and therapy [10,12,13,14,15,16,29].

Furthermore, our analyses indicate that NAD+ metabolism can be an important source of SCC diagnostic biomarkers:

(i) several differentially expressed NMRGs display good diagnostic power in discriminating tumors from control specimens. Specifically, we found 6 genes for HNSCC (*AOX1*, *NMNAT2*, *ENPP3*, *NADK*, *CD38*, and *PARP1*), 11 genes for LuSCC (*AOX1*, *NNMT*, *NMNAT2*, *NMNAT3*, *ENPP2*, *ENPP3*, *NMRK2*, *NADSYN1*, *SIRT1*, *CD38*, and *PARP1*) and 11 genes for CeSCC (*AOX1*, *NMNAT3*, *ENPP1*, *ENPP2*, *ENPP3*, *NMRK2*, *PNP*, *NADSYN1*, *SIRT1*, *SIRT3*, and *CD38*);
(ii) three differentially-expressed NMRGs can be considered relevant SCC-type specific biomarkers;
(iii) specific NMRG signatures display high diagnostic power for several clinical-pathological characteristics.

The identification of these biomarkers could also give insight into common or specific pathogenetic mechanisms. In fact, most differentially expressed NMRG biomarkers are common for two or three SCCs. *AOX1*, *ENPP3*, and *NMRK2* were down-modulated in all analyzed tumor specimens, whereas *PARP1* was up-regulated. *AOX1* down-regulation in all SCCs underlines its potential importance in SCC pathogenesis. *AOX1* encodes aldehyde oxidase 1 that is down-regulated in several tumors mainly as a consequence of hypermethylation [30,31,32]. This gene is implicated in the AKT pathway as well as NAD+ catabolism. For instance, decreased AOX1 activity can allow the accumulation of the MNAM catabolite. Notably, MNAM is metabolically active and can regulate posttranslational protein acetylation through Sirt1 modulation [33]. *PARP1* up-regulation is a common feature of analyzed SCC. The enzyme has an important role in the repair of single-strand breaks, the most common type of DNA damage, and is a well-established therapeutic target for several tumors, including HNSCC. PARP inhibitors increase DNA damage, enhance immune priming, and induce adaptive upregulation of PD-L1 expression [34]. *ENPP3* and *NMRK2* encode enzymes that catalyze the biosynthesis of NAM mononucleotide (NAMN). These enzymes are up-regulated in colorectal and renal cancers representing specific biomarkers and therapeutic targets [35,36,37]. Thus, *ENPP3* and *NMRK2* down-regulation in all analyzed SCCs can strengthen the role of NAM replenishment in SCC prevention. Significant down-regulation of *AOX1* and *ENPP3* and up-regulation of *PARP1* are also observed in cutaneous SCC (data from GSE datasets). Data regarding *NMRK2* are not available. Therefore, the common modulation of these three genes can play a key role in SCC pathogenesis.

Between differentially expressed NMRGs, *NADK* up-regulation resulted in an HNSCC-specific diagnostic biomarker. *NADK* up-regulation can be due to gene mutations or activated oncogenic signaling [38,39]. NADK catalyzes the phosphorylation of NAD+ to NADP+ using ATP. The reduced form NADPH is high in proliferating tumor cells and acts as a cofactor for the synthesis of macromolecules as well as ROS scavengers [39]. Thionicotinamide and other NAM analogs are able to inhibit NADK by targeting its NAD+ binding site. They sensitize cells to chemotherapeutic agents by increasing ROS levels and inhibiting NADPH-dependent synthetic pathways [40].

*NNMT* down-regulation resulted in a LuSCC-specific diagnostic biomarker. *NNMT* is an intracellular methyltransferase that catalyzes N-methylation of NAM to form MNAM, in which S-adenosyl-L-methionine (SAM) is the methyl donor. MNAM is eventually excreted from the body. A growing body of evidence indicates that, beyond clearance of excess vitamin B3, NNMT is implicated in the regulation of multiple metabolic pathways in tumor cells by remodeling cellular epigenetic states and generating active metabolites. As *NNMT* has been found up- or down-regulated in different tumors, it seems to play a complex role in cancer progression and function in a tissue-specific manner [33,41,42,43]. The observed low expression of *NNMT* in LuSCC TCGA samples can induce an unbalance of NAM and SAM levels and, in turn, affect NAD+-dependent redox reactions as well as the regulation of the epigenetic landscape. For instance, the knockdown of *NNMT* in tumor cell lines increases the SAM/SAH ratio and global H3K9 and H3K27 trimethylation. Other molecules in addition to histones can be differentially methylated by changes in *NNMT* expression and SAM levels [41].

*ENPP1* down-regulation resulted in a CeSCC-specific diagnostic biomarker. ENPP1 participates in the hydrolysis of different purine nucleotides in several processes, is frequently overexpressed in tumors, and is associated with poor prognosis and survival. ENPP1 promotes an immunosuppressive tumor microenvironment by the imbalance of ATP/adenosine and impairs the STING (stimulator of interferon genes) pathway immune response by the hydrolysis of the effector cGMP–AMP [44]. Thus, *ENPP1* down-regulation can be unfavorable for creating an immunosuppressive landscape in CeSCC but could foster a strong interferon-mediated immune response.

Although further in vitro and in vivo experiments will be needed, *AOX1* can be a potential common therapeutic target for SCC treatment, whereas *NADK*, *NNMT*, and *ENPP1* can be for HNSCC, LuSCC, and CeSCC, respectively.

The current therapeutic SCC treatments are mostly based on the TNM staging system classification, leading to quite homogeneous treatment options for patients. Our bioinformatic analyses identified specific NMRG signatures that display high diagnostic power for discriminating patients on the basis of several clinical-pathological characteristics such as stage, grade, or HPV infection. Thus, the validation of these signatures may be helpful for diagnosis and personalized treatments.

The manipulation of NAD+ bioavailability represents a promising therapeutic strategy [10,12,29]. Preclinical studies indicated that NAM administration is tumor suppressive by disrupting several processes such as proliferation, apoptosis, invasion, and metastasis. Moreover, NAM administration can re-establish sensitivity to therapy in chemoresistant forms, inhibit T cell exhaustion, and increase T cell differentiation, and have important implications in immunotherapy [12,26]. The role of NAD+ in immunomodulation is context- and signaling-pathway-specific [26]. Our bioinformatic analyses indicate a potential SCC-type specific role of NMRGs in the tumor immune microenvironment changes. Indeed, the correlation between NMRG levels and TII cells or suppressive immune milieu is mainly observed in HNSCC samples. The lowest association is observed in CeSCCs.

Furthermore, the evaluation of the overall survival, based on NMRG expression levels in patients from the TCGA dataset, identified potential prognostic biomarkers or therapeutic targets. High-risk HNSCC patients are characterized by high levels of *NNMT*, *NAMPT*, or *PNP*. Notably, a signature based on these three genes displays good prognostic power. *NNMT* and *NAMPT* levels positively correlated with TII cells in the microenvironment, different from *PNP*. An *NNMT* increase is significantly associated with poor prognosis and immune suppressive microenvironment in many tumors [45]. We found that the high-risk group with high *NNMT* levels displays a significant increase in *PD-1* and *PD-L1*. Also, *NAMPT* up-regulation is correlated with unfavorable overall survival in several tumors. NAMPT-mediated signaling plays a key role in tumor progression by modulating proliferation, cell plasticity, cytokine secretion, angiogenesis, metastasis, and in microenvironment modifications [46]. Indeed, NAMPT drives INFγ-induced PD-L1 expression and induces tumor immune evasion in a CD8+ T cell-dependent manner [28]. Moreover, *NAMPT* overexpression effects can also confer chemoresistance to anticancer drugs [46]. In keeping with the literature data, we found that the high-risk group of patients with high *NAMPT* levels shows a significant increase in *PD-L1*. The high-risk group with high *PNP* expression displays decreased levels of *PD-1* and a slight increase of *PD-L1*. Up to now, data concerning an association between *PNP* increase and tumor survival have not been reported. However, the enzyme purine nucleoside phosphorylase may play a role in cancer chemoresistance by catalyzing the degradation of potentially cytotoxic purine analogs [47]. 

High-risk LuSCC patients are characterized by low levels of *NADSYN1*. Its levels negatively correlated with TII cells and no significant difference in *PD-1* or *PD-L1* expression was observed between high- and low-risk groups. Up to now, no data have been reported regarding *NADSYN1* and tumor survival. NAD synthetase is an enzyme for NAD+ biosynthesis from tryptophane and, thus, is involved in NAD+ replenishment. Notably, NAM administration is able to suppress lung tumor formation in animal models [12].

High-risk CeSCC patients are characterized by high levels of *AOX1* and *NMNAT1*. Of note, a signature based on these two NMRGs displays a good prognostic power. The expression level of *AOX1* and *NMNAT1* correlates with the infiltration of a few TII cell types. The high-risk group with high AOX1 levels displays a significant increase in *PD-1* expression. No significant difference in *PD-1* or *PD-L1* expression was observed between high- and low-risk groups expressing *NMNAT1*. High levels of *AOX1* are associated with better prognosis of bladder, renal, and pancreatic cancers [31,39], whereas high *NMNAT1* expression is associated with poor survival in cancer patients with hepatocellular carcinoma [48]. Although *NNMT1*, *NAMPT*, and *AOX1* are well-studied enzymes in several tumors, *PNP*, *NADSYN*, and *NMNAT1* stand out as additional understudied enzymes that justify further validation as therapeutic targets.

## 5. Conclusions

Our study is focused on the bioinformatic investigation of an NMRG profile as a source of potential SCC biomarkers and therapeutic targets.

Beyond the well-established tumor marker *PARP1*, *AOX1* can be a potential common therapeutic target for SCC treatment. The hypermethylation of the gene suggests that epigenetic drugs can be a promising tool to be assayed. The down-regulation of *ENPP3* and *NMRK2*, found in all SCCs, can reduce NMAN bioavailability. Our findings agree with SCC preclinical and clinical studies using NAD+ precursors underlying their importance as a drug for chemoprevention and adjuvant therapy. Furthermore, the use of NAM analogs can be a promising strategy for synergizing with chemotherapy by *NADK* inhibition.

Therefore, future experimental studies concerning identified NMRGs and their validation on different populations may provide additional insights into pathogenetic mechanisms as well as improve the therapeutic choice.

## Figures and Tables

**Table 1:** Correlation of NMRG expression with immune infiltrates and checkpoints. Red spots indicate a significant positive correlation, blue spots indicate a significant negative correlation and grey spots indicate no correlation. Data obtained with genes displaying low or undetectable expression in tumor samples are in grey.

	HNSCC								LuSCC								CeSCC							
Genes	B Cell	CD8+ T Cell	CD4+ T Cell	Macrophage	Neutrophil	Dendritic Cell	PD-1	PD-L1	B Cell	CD8+ T Cell	CD4+ T Cell	Macrophage	Neutrophil	Dendritic Cell	PD-1	PD-L1	B Cell	CD8+ T Cell	CD4+ T Cell	Macrophage	Neutrophil	Dendritic Cell	PD-1	PD-L1
**AOX1**	0.24	0.13	0.41	0.30	0.23	0.32	0.12	0.17	0.32	0.34	0.35	0.45	0.40	0.50	0.31	0.10	0.18	−0.02	0.11	0.13	0.09	0.08	−0.04	0.00
**NNMT**	0.05	0.10	0.25	0.45	0.27	0.32	0.08	0.14	0.17	0.29	0.27	0.47	0.48	0.48	0.18	0.01	−0.05	0.01	−0.14	0.23	0.06	−0.03	−0.01	−0.08
**NAMPT**	−0.08	0.06	0.10	0.08	0.11	0.12	0.01	0.25	−0.09	0.05	−0.27	0.03	0.03	−0.06	−0.08	0.38	−0.11	0.05	−0.12	−0.19	0.18	0.20	−0.08	0.26
**NMNAT1**	0.06	0.02	0.31	0.15	0.22	0.20	0.20	0.12	0.06	0.10	0.16	0.21	0.27	0.18	−0.02	−0.12	0.05	−0.02	0.12	0.08	0.06	0.03	−0.07	0.00
**NMNAT2**	0.04	−0.05	0.29	0.12	0.12	0.21	−0.05	0.10	0.02	0.13	0.33	0.24	0.27	0.28	0.13	−0.09	−0.03	−0.07	−0.04	−0.04	0.02	−0.09	−0.09	−0.09
**NMNAT3**	0.18	0.03	0.14	0.07	−0.15	−0.04	0.05	−0.17	0.00	−0.15	−0.08	−0.15	−0.26	−0.23	−0.09	0.07	0.13	0.00	0.14	−0.02	−0.07	0.08	−0.02	0.02
**ENPP1**	0.27	0.20	0.35	0.48	0.13	0.40	0.12	0.07	0.17	0.18	0.21	0.20	0.15	0.28	0.19	0.06	0.17	−0.01	−0.01	0.30	−0.08	−0.16	−0.11	−0.21
**ENPP2**	0.41	0.50	0.65	0.73	0.57	0.75	0.56	0.36	0.42	0.48	0.39	0.58	0.49	0.66	0.43	0.26	0.30	0.41	0.34	0.42	0.34	0.41	0.33	0.17
**ENPP3**	0.46	0.35	0.35	0.30	0.17	0.34	0.32	0.11	0.26	0.24	0.19	0.26	0.28	0.24	0.18	0.07	0.15	0.12	0.07	0.21	−0.03	−0.13	0.03	−0.22
**NMRK1**	0.06	0.16	0.19	0.16	0.18	0.17	0.26	0.14	0.00	0.03	−0.14	−0.02	−0.15	−0.09	0.00	0.27	−0.13	0.06	0.03	−0.03	0.05	0.14	0.00	0.12
**PNP**	−0.29	−0.19	−0.15	−0.15	0.01	−0.12	−0.22	0.13	−0.13	−0.10	−0.04	0.07	0.09	0.05	−0.11	−0.02	−0.15	−0.07	−0.10	−0.11	0.03	0.05	−0.19	0.09
**NADK**	0.11	0.17	0.40	0.26	0.36	0.33	0.30	0.25	0.08	0.03	0.41	0.14	0.17	0.25	0.25	0.08	0.09	0.05	0.17	0.11	0.15	0.09	0.03	0.03
**NADSYN1**	0.02	−0.05	0.04	0.01	−0.06	−0.06	−0.06	−0.02	−0.01	−0.18	0.14	−0.11	−0.12	−0.11	0.06	0.06	0.00	−0.09	0.22	−0.02	0.06	0.04	−0.02	0.10
**SIRT1**	0.20	0.15	0.45	0.28	0.25	0.34	0.21	0.27	0.25	0.28	0.37	0.30	0.27	0.36	0.28	0.03	0.01	0.11	0.04	0.03	0.03	−0.06	−0.04	−0.01
**SIRT3**	−0.13	−0.10	−0.15	−0.07	0.02	−0.07	−0.04	−0.11	−0.06	−0.09	0.04	−0.02	−0.16	−0.07	−0.08	−0.08	−0.10	0.06	−0.14	0.04	−0.08	−0.09	0.06	−0.14
**CD38**	0.27	0.35	0.35	0.31	0.37	0.42	0.41	0.27	0.47	0.42	0.24	0.28	0.35	0.45	0.55	0.31	0.29	0.45	0.38	0.11	0.49	0.56	0.55	0.34
**PARP1**	0.24	0.19	0.39	0.28	0.19	0.30	0.26	0.18	0.10	0.01	0.18	−0.11	−0.01	0.07	0.20	0.19	0.19	0.10	0.16	0.03	0.13	0.15	0.06	0.10

**Table 2:** Correlation data between NMRGs and immune checkpoint expression. * *p* ≤ 0.05, ** *p* ≤ 0.01, **** *p* ≤ 0.0001, ns = no significance.

			Pearson r	R Squared	*p* Value	Significance	N. of Samples
**HNSCC**	**NNMT**	**HIGH vs. PD-1**	0.06658	0.004433	0.2934	ns	251
		**HIGH vs. PD-L1**	−0.01235	0.0001526	0.8456	ns	251
		**LOW vs. PD-1**	0.06059	0.003671	0.3371	ns	253
		**LOW vs. PD-L1**	0.1235	0.01525	0.0497	*	253
	**NAMPT**	**HIGH vs. PD-1**	−0.1952	0.03812	0.0026	**	235
		**HIGH vs. PD-L1**	−0.08881	0.007886	0.1749	ns	235
		**LOW vs. PD-1**	0.0748	0.005595	0.2214	ns	269
		**LOW vs. PD-L1**	0.2659	0.07072	<0.0001	****	269
	**PNP**	**HIGH vs. PD-1**	−0.06807	0.004634	0.2677	ns	267
		**HIGH vs. PD-L1**	0.0304	0.0009239	0.621	ns	267
		**LOW vs. PD-1**	−0.1378	0.01899	0.034	*	237
		**LOW vs. PD-L1**	0.1493	0.02228	0.0215	*	237
**LuSCC**	**NADSYN1**	**HIGH vs. PD-1**	0.03129	0.0009792	0.6354	ns	232
		**HIGH vs. PD-L1**	−0.02539	0.0006447	0.7005	ns	232
		**LOW vs. PD-1**	0.06948	0.004827	0.2597	ns	265
		**LOW vs. PD-L1**	0.1615	0.02609	0.0084	**	265
**CeSCC**	**NMNAT1**	**HIGH vs. PD-1**	−0.09107	0.008294	0.2293	ns	176
		**HIGH vs. PD-L1**	−0.08989	0.00808	0.2354	ns	176
		**LOW vs. PD-1**	0.1255	0.01575	0.1548	ns	130
		**LOW vs. PD-L1**	0.07924	0.006279	0.3702	ns	130
	**AOX1**	**HIGH vs. PD-1**	0.009421	0.00008875	0.9208	ns	114
		**HIGH vs. PD-L1**	0.1465	0.02146	0.1199	ns	114
		**LOW vs. PD-1**	−0.03515	0.001236	0.6284	ns	192
		**LOW vs. PD-L1**	−0.1138	0.01295	0.116	ns	192

## Data Availability

The datasets generated and/or analyzed during the current study are available in the TCGA and GTEx repositories, available at links clearly indicated in the manuscript.

## Supplementary Materials

The following supporting information can be downloaded at: https://www.mdpi.com/article/10.3390/cancers16020309/s1, Figure S1. NMRGs with significant differential gene expression levels in age and gender groups of SCC patients. Violin plots show the significant differential expression of NMRGs between age-related groups (A) and (B) gender groups in HNSCC (red); LuSCC (blue) and CeSCC (green). HNSCC: head and neck squamous cell carcinoma; LuSCC: lung squamous cell carcinoma; CeSCC: cervix squamous cell carcinoma. Figure S2. Modulation of different NMRG expressions based on SCC types. HNSCC: head and neck squamous cell carcinoma; LuSCC: lung squamous cell carcinoma; CeSCC: cervix squamous cell carcinoma. Figure S3. AUC comparison for the 18 NMRGs based on SCC type. HNSCC: head and neck squamous cell carcinoma; LuSCC: lung squamous cell carcinoma; CeSCC: cervix squamous cell carcinoma. Figure S4. Overall survival for HNSCCs and CeSCCs using a combination of specific NMRGs signatures increased the prognostic significance with respect to single NMRGs. HNSCC: head and neck squamous cell carcinoma; CeSCC: cervix squamous cell carcinoma. Table S1. Clinicopathologic characteristics of patients. Table S2. Receiver Operating Characteristic (ROC) analysis on Head and Neck squamous cell carcinoma (HNSCC) specimens. Table S3. Receiver Operating Characteristic (ROC) analysis on Lung squamous cell carcinoma (LuSCC) specimens. Table S4. Receiver Operating Characteristic (ROC) analysis on Cervix squamous cell carcinoma (CeSCC) specimens. Table S5. Survival data.

## List of Abbreviations

SCC	Squamous cell carcinoma
HNSCC	Head and Neck Squamous cell carcinoma
LuSCC	Head and Neck Squamous cell carcinoma
CeSCC	cervix Squamous cell carcinoma
HPV	human papillomavirus
NSCLC	non-small cell lung cancer
NAD+	Nicotinamide adenine dinucleotide
NAM	nicotinamide
NMN	nicotinamide mononucleotide
NR	nicotinamide riboside
NMRG	NAD+ metabolism-related gene
TCGA	The Cancer Genome Atlas
GTEx	Genotype-Tissue Expression
ROC	Receiver Operating Characteristic
AUC	Area under the curve
PCA	Principal component analysis
KIRC	Kidney Renal clear cell Carcinoma
GBM	Glioblastoma
SARC	Sarcoma
TIMER	Tumor IMmune Estimation Resource
OS	overall survival
SAM	S-adenosyl-L-methionine
ATP	adenosine triphosphate
NAMPT	NAM phosphoribosyltransferase
NAMN	NAM mononucleotide
NMNATs	NMN adenyltransferases
NADK	NAD kinase
cADPRS	cyclic ADP-ribose synthase
PARPs	Poly-ADP-ribose-polymerases
SIRT	Sirtuins
Sub	substrates
MNA	1-methyl-NAM
NNMT	NAM-N-methyltransferase
2-Pyr	l-methyl-2-pyridone-5-carboxamide
4-Pyr	l-methyl-4-pyridone- 5-carboxamide
