# Supplementary material for: NAD+ Metabolism-Related Gene Profile Can Be a Relevant Source of Squamous Cell Carcinoma Biomarkers
Source: Cancers (Basel). 2024 Jan 11;16(2):309. doi: 10.3390/cancers16020309 (PMC10814490; doi:10.3390/cancers16020309)
Supplement: Supplementary file 1 [file cancers-16-00309-s001.zip › Figure S3_new.pdf]

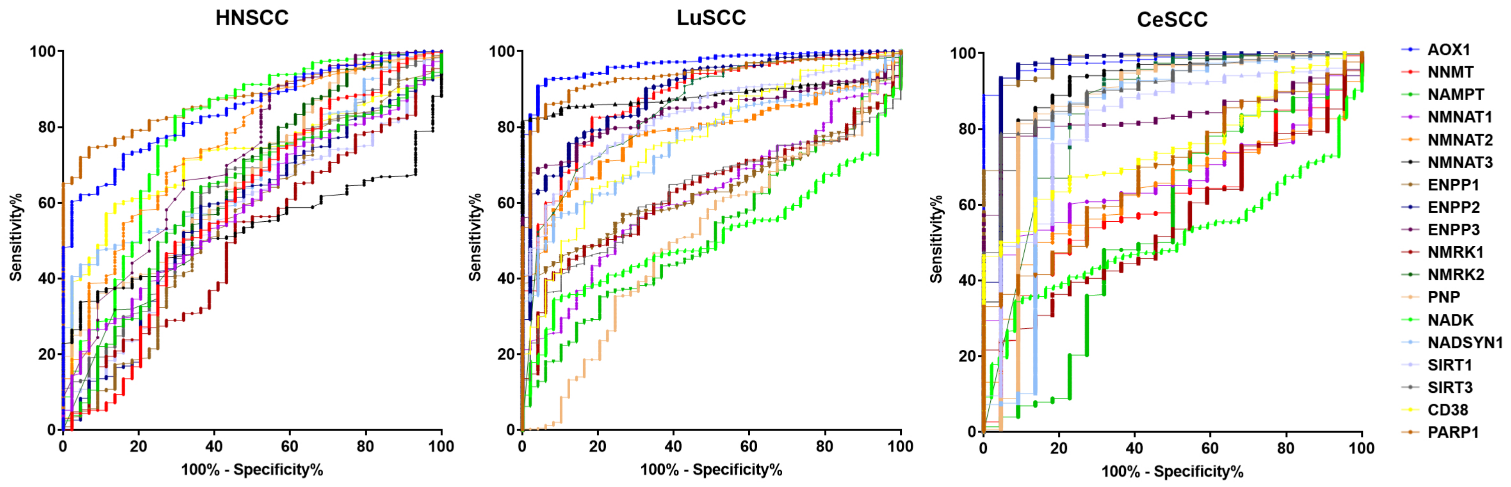
HNSCC
LuSCC
CeSCC
Sensitivity%
100% - Specificity%
0
20
40
60
80
100
AOX1
NNMT
NAMPT
NMNAT1
NMNAT2
NMNAT3
ENPP1
ENPP2
ENPP3
NMRK1
NMRK2
PNP
NADK
NADSYN1
SIRT1
SIRT3
CD38
PARP1
