# Supplementary material for: NAD+ Metabolism-Related Gene Profile Can Be a Relevant Source of Squamous Cell Carcinoma Biomarkers
Source: Cancers (Basel). 2024 Jan 11;16(2):309. doi: 10.3390/cancers16020309 (PMC10814490; doi:10.3390/cancers16020309)
Supplement: Supplementary file 1 [file cancers-16-00309-s001.zip › Supplemental Tables.pdf]

Supplemental Table S1. Clinicopathologic characteristics of patients.

			HNSCC (N=44; T=504)	LuSCC (N=49; T=501)	CeSCC (N= 3; T= 306)
Age	Normal	mean (sd)	62.11 (13.57)	68.63 (8.49)	54.33 (14.01)
		median (IQR)	64 (53.25 - 68.75)	69 (62.5 - 74.5)	55 (40 - 68)
	Tumor	mean (sd)	61.05 (11.89)	67.2 (8.58)	48.18 (13.82)
		median (IQR)	61 (53 - 69)	68 (62 - 73)	46 (38 - 56.25)
Sex	Normal	F	31.80%	28.6%	100%
		M	68.2%	71.4%	0%
	Tumor	F	26.6%	26%	100%
		M	73.4%	74%	0%
Race	Normal	white	93%	95%	NA
		black or african american	4.7%	5%	NA
		asian	2.3%	/	NA
	Tumor	white	87.7%	90%	77.7%
		black or african american	9.6%	7.7%	11.5%
		asian	2%	2.3%	7.4%
		american indian or alaska native	0.4%	/	2.6%
		native hawaiian or other pacific islander	/	/	0.7%
Stage	Tumor	I	5%	49%	53%
		II	14%	32%	22%
		III	16%	17%	15%
		IV	52%	1%	7%
Grade	Tumor	I	12%	NA	6%
		II	60%	NA	44%
		III	24%	NA	39%
		IV	0.40%	NA	0.30%
HPV status detection (n=83)	Tumor	HPV_negative	77%	NA	NA
		HPV_positive	23%	NA	NA
Anatomic site	Tumor	oral cavity	66%	/	/
		oropharinx	34%	/	/

Supplemental Table S2. Receiver Operating Characteristic (ROC) analysis on Head and Neck squamous cell carcinoma (HNSCC) specimens. AUC values with their significance for each gene in discriminating tumor vs normal tissue, stage, grade, anatomic site and HPV infection status.

	Normal vs Tumor		stage 1 vs stage 2		stage 1 vs stage 3		stage 1 vs stage 4		G1 vs G2		G1 vs G3		oropharix vs oral cavity		hpv status	
**Genes**	**AUC**	**p value**	**AUC**	**p value**	**AUC**	**p value**	**AUC**	**p value**	**AUC**	**p value**	**AUC**	**p value**	**AUC**	**p value**	**AUC**	**p value**
**AOX1**	**0.8461**	**<0.0001**	**0.6466**	**0.0302**	**0.6625**	**0.0146**	**0.7012**	**0.0009**	0.5751	0.0626	0.585	0.0609	0.5364	0.2161	0.6086	0.1525
**NNMT**	0.5912	0.0448	0.5657	0.3311	0.5289	0.6646	0.5731	0.2275	0.563	0.1179	0.6237	0.0063	**0.5793**	**0.007**	**0.7936**	**0.0001**
**NAMPT**	0.6149	0.0114	0.512	0.8591	0.5544	0.4135	0.5217	0.7202	**0.6388**	**0.0006**	0.5854	0.0597	0.5598	0.0422	**0.7229**	**0.0033**
**NMNAT1**	0.5901	0.0472	0.5409	0.5457	0.5448	0.5008	0.5068	0.9106	0.5547	0.1748	**0.6279**	**0.0048**	0.5493	0.094	**0.7771**	**0.0003**
**NMNAT2**	**0.7561**	**<0.0001**	0.5371	0.5828	0.578	0.2414	0.5502	0.4074	0.519	0.6375	0.5767	0.0906	0.5371	0.2076	0.5337	0.6568
**NMNAT3**	0.532	0.4806	0.5146	0.8294	0.6	0.133	0.6031	0.0884	0.5787	0.0509	0.5741	0.102	**0.6812**	**<0.0001**	0.6813	0.0168
**ENPP1**	0.5522	0.2505	0.5043	0.9495	0.5084	0.9001	0.5515	0.3946	**0.6159**	**0.004**	**0.6664**	**0.0002**	0.5272	0.3557	0.5913	0.2289
**ENPP2**	0.5721	0.1123	0.5763	0.2592	0.601	0.1291	0.5647	0.2855	0.6034	0.0103	**0.6822**	**<0.0001**	0.5332	0.2595	0.5748	0.324
**ENPP3**	**0.722**	**<0.0001**	**0.6786**	**0.0083**	**0.6509**	**0.0234**	0.6175	0.0523	0.5975	0.0156	**0.6337**	**0.0032**	**0.6165**	**<0.0001**	0.6591	0.036
**NMRK1**	0.5033	0.9418	0.5994	0.1414	0.5782	0.2399	**0.6395**	**0.0212**	0.5132	0.7437	0.569	0.1281	0.5126	0.6693	**0.7175**	**0.0041**
**NMRK2**	0.6455	0.0014	0.6303	0.054	0.6258	0.0587	**0.6395**	**0.0212**	0.5159	0.693	0.5358	0.4291	**0.61**	**0.0002**	0.6328	0.08
**PNP**	0.674	0.0001	0.5194	0.7738	0.5757	0.2554	0.5239	0.6931	0.5028	0.9449	**0.636**	**0.0027**	0.543	0.1437	**0.7747**	**0.0003**
**NADK**	**0.7872**	**<0.0001**	0.5574	0.3957	0.5377	0.5709	0.5545	0.3679	0.5688	0.0879	0.6126	0.013	0.5075	0.7986	0.5711	0.3485
**NADSYN1**	0.6866	<0.0001	0.5431	0.5234	0.5403	0.5453	0.5126	0.8352	0.524	0.5511	0.507	0.8765	**0.5931**	**0.0015**	0.6254	0.0983
**SIRT1**	0.5569	0.2101	0.5449	0.507	0.5701	0.2921	0.514	0.8165	**0.6168**	**0.0038**	**0.691**	**<0.0001**	0.5449	0.1266	0.6349	0.0755
**SIRT3**	0.6249	0.006	**0.6851**	**0.0062**	**0.681**	**0.0065**	**0.6722**	**0.0044**	0.5809	0.0448	0.6164	0.0102	0.5349	0.2356	0.6016	0.1807
**CD38**	**0.7182**	**<0.0001**	0.5266	0.6943	0.517	0.7989	0.5447	0.4607	0.5249	0.5374	0.5554	0.222	0.5541	0.066	0.5633	0.4039
**PARP1**	**0.8737**	**<0.0001**	0.5966	0.1532	0.5511	0.4423	**0.6194**	**0.0486**	**0.7114**	**<0.0001**	**0.8035**	**<0.0001**	**0.6191**	**<0.0001**	**0.7697**	**0.0004**

Data obtained with genes displaying low or undetectable expression in tumor samples are in grey.

Supplemental Table S3. Receiver Operating Characteristic (ROC) analysis on Lung squamous cell carcinoma (LuSCC) specimens.
AUC values with their significance for each gene in discriminating tumor vs normal tissue and stage.

	Normal vs Tumor		stage 1 vs stage 2		stage 1 vs stage 3		stage 1 vs stage 4	
**Genes**	**AUC**	**p value**	**AUC**	**p value**	**AUC**	**p value**	**AUC**	**p value**
**AOX1**	**0.9681**	**<0.0001**	0.513	0.6568	0.5003	0.9936	0.5659	0.5525
**NNMT**	**0.8766**	**<0.0001**	0.5502	0.0867	0.5524	0.1516	0.6212	0.2744
**NAMPT**	0.5257	0.5523	0.5123	0.6747	0.5038	0.9182	0.572	0.516
**NMNAT1**	0.6203	0.0054	0.546	0.1161	0.5151	0.6792	0.5161	0.8845
**NMNAT2**	**0.7804**	**<0.0001**	0.5419	0.1529	0.5031	0.9314	0.627	0.2519
**NMNAT3**	**0.8829**	**<0.0001**	0.5068	0.8153	0.5118	0.7463	0.6142	0.3032
**ENPP1**	0.6353	0.0018	0.5478	0.1028	0.5714	0.0511	0.5547	0.6215
**ENPP2**	**0.8903**	**<0.0001**	0.5197	0.5014	0.5197	0.5904	0.5246	0.8245
**ENPP3**	**0.8383**	**<0.0001**	0.5923	0.0016	0.5225	0.5377	0.524	0.8286
**NMRK1**	0.6307	0.0025	0.5114	0.6966	0.5237	0.5168	0.5404	0.7156
**NMRK2**	**0.8474**	**<0.0001**	0.5507	0.0833	0.5512	0.1613	0.534	0.7594
**PNP**	0.5128	0.7671	0.5319	0.2765	0.5312	0.3936	0.6382	0.2127
**NADK**	0.5129	0.766	0.5441	0.1319	0.5603	0.099	0.589	0.4222
**NADSYN1**	**0.7605**	**<0.0001**	0.5313	0.2858	0.5582	0.1114	0.5954	0.3894
**SIRT1**	**0.8303**	**<0.0001**	0.5167	0.5687	0.5354	0.3325	0.5211	0.8492
**SIRT3**	0.6272	0.0033	0.5144	0.6228	0.5107	0.7697	0.6575	0.1555
**CD38**	**0.7674**	**<0.0001**	0.5361	0.2183	0.5676	0.0647	0.5512	0.6441
**PARP1**	**0.9341**	**<0.0001**	0.5491	0.094	0.5315	0.3884	0.5504	0.6498

Data obtained with genes displaying low or undetectable expression in tumor samples are in grey.

Supplemental Table S4. Receiver Operating Characteristic (ROC) analysis on Cervix squamous cell carcinoma (CeSCC) specimens.
AUC values with their significance for each gene in discriminating tumor vs normal tissue, grade and histological type.

	Normal vs Tumor		G1 vs G 2		G1 vs G3		Adenocarcinoma vs SCC	
**Genes**	**AUC**	**p value**	**AUC**	**p value**	**AUC**	**p value**	**AUC**	**p value**
**AOX1**	**0.9798**	**<0.0001**	0.5185	0.7941	0.5376	0.5994	0.5279	0.5231
**NNMT**	0.6069	0.094	0.5439	0.5365	0.55	0.485	0.517	0.6977
**NAMPT**	0.5432	0.4989	0.5099	0.8886	0.5104	0.8845	**0.6655**	**0.0002**
**NMNAT1**	0.6693	0.008	0.5415	0.5585	0.5484	0.4986	0.5271	0.5343
**NMNAT2**	0.6365	0.0324	0.5515	0.4683	0.5024	0.9729	**0.6998**	**<0.0001**
**NMNAT3**	**0.9202**	**<0.0001**	0.5458	0.5186	0.5345	0.6298	**0.7581**	**<0.0001**
**ENPP1**	**0.9715**	**<0.0001**	0.6096	0.1226	0.5856	0.2318	**0.8304**	**<0.0001**
**ENPP2**	**0.9646**	**<0.0001**	0.5924	0.1929	0.5378	0.5973	0.5167	0.7015
**ENPP3**	**0.8376**	**<0.0001**	0.6212	0.0875	0.6163	0.1041	**0.8102**	**<0.0001**
**NMRK1**	0.5525	0.4106	0.5887	0.2113	0.5867	0.2258	**0.6573**	**0.0003**
**NMRK2**	**0.85**	**<0.0001**	0.6008	0.1556	0.6415	0.048	0.5082	0.8504
**PNP**	**0.8622**	**<0.0001**	0.5563	0.4273	0.6053	0.1414	0.5052	0.9055
**NADK**	0.5541	0.3962	0.5281	0.6924	0.5203	0.7762	**0.6487**	**0.0007**
**NADSYN1**	**0.8217**	**<0.0001**	0.5462	0.515	0.5057	0.936	0.564	0.1429
**SIRT1**	**0.8164**	**<0.0001**	0.5004	0.9956	0.5217	0.7621	**0.6917**	**<0.0001**
**SIRT3**	**0.9072**	**<0.0001**	0.5092	0.8973	0.5203	0.7762	**0.7019**	**<0.0001**
**CD38**	**0.7539**	**<0.0001**	0.5341	0.6307	0.5396	0.5802	**0.6889**	**<0.0001**
**PARP1**	0.691	0.0028	0.5004	0.9956	0.5741	0.3007	0.5134	0.7592

Data obtained with genes displaying low or undetectable expression in tumor samples are in grey.

Supplemental Table S5. Survival data.

	HNSCC (504 pt)			LuSCC (497)			CeSCC (306 pt)		
**Genes**	**n. of patients with low exp**	**n. of patients with high exp**	**p value**	**n. of patients with low exp**	**n. of patients with high exp**	**p value**	**n. of patients with low exp**	**n. of patients with high exp**	**p value**
**AOX1**	296	208	0.448 L	269	228	0.4193 H	194	112	**0.0389 H**
**NNMT**	253	251	**0.0002 H**	239	258	0.2632 H	166	140	0.0676 H
**NAMPT**	269	235	**0.012 H**	280	217	0.351 H	159	147	0.1698 H
**NMNAT1**	246	258	0.871	261	236	0.7233	130	176	**0.0106 H**
**NMNAT2**	294	210	0.2343 L	341	156	0.7506 L	193	113	0.1565 H
**NMNAT3**	275	229	0.9994 H	254	243	0.9034	142	164	0.1661 L
**ENPP1**	300	204	0.4581 H	291	206	0.4945 L	192	114	0.6788 H
**ENPP2**	256	248	0.6401 H	238	259	0.1419 H	149	157	0.8398 L
**ENPP3**	359	145	**0.0201 L**	328	169	0.6303	239	67	0.6813 L
**NMRK1**	241	263	0.1921 H	261	236	0.3796 L	146	160	0.4697 L
**NMRK2**	346	158	0.3701 H	358	139	0.3383 H	193	113	0.1678 H
**PNP**	237	267	**0.0365 H**	261	236	0.4369 H	149	157	0.0839 H
**NADK**	236	268	0.6067 H	239	258	0.0702	137	169	0.3037 H
**NADSYN1**	297	207	0.8463 H	265	232	**0.0445 L**	159	147	0.6174 L
**SIRT1**	228	276	0.796 H	247	250	0.4624 H	135	171	0.7741
**SIRT3**	249	255	0.3315 L	255	242	0.1358 L	165	141	0.5453 L
**CD38**	247	257	0.2699 L	243	254	0.3066 H	161	145	0.642
**PARP1**	245	259	0.5768 H	252	245	0.1769 L	142	164	0.3193 H

H letter indicates lower survival associated with high NMRG expression level; L letter indicates lower survival associated with low NMRG gene expression level. p value ≤ 0.05 are in bold. Data obtained with genes displaying low or undetectable expression in tumor samples are in grey.
