# Supplementary figures and images for: NAD+ Metabolism-Related Gene Profile Can Be a Relevant Source of Squamous Cell Carcinoma Biomarkers
Source: Cancers (Basel). 2024 Jan 11;16(2):309. doi: 10.3390/cancers16020309 (PMC10814490; doi:10.3390/cancers16020309)

**(A)**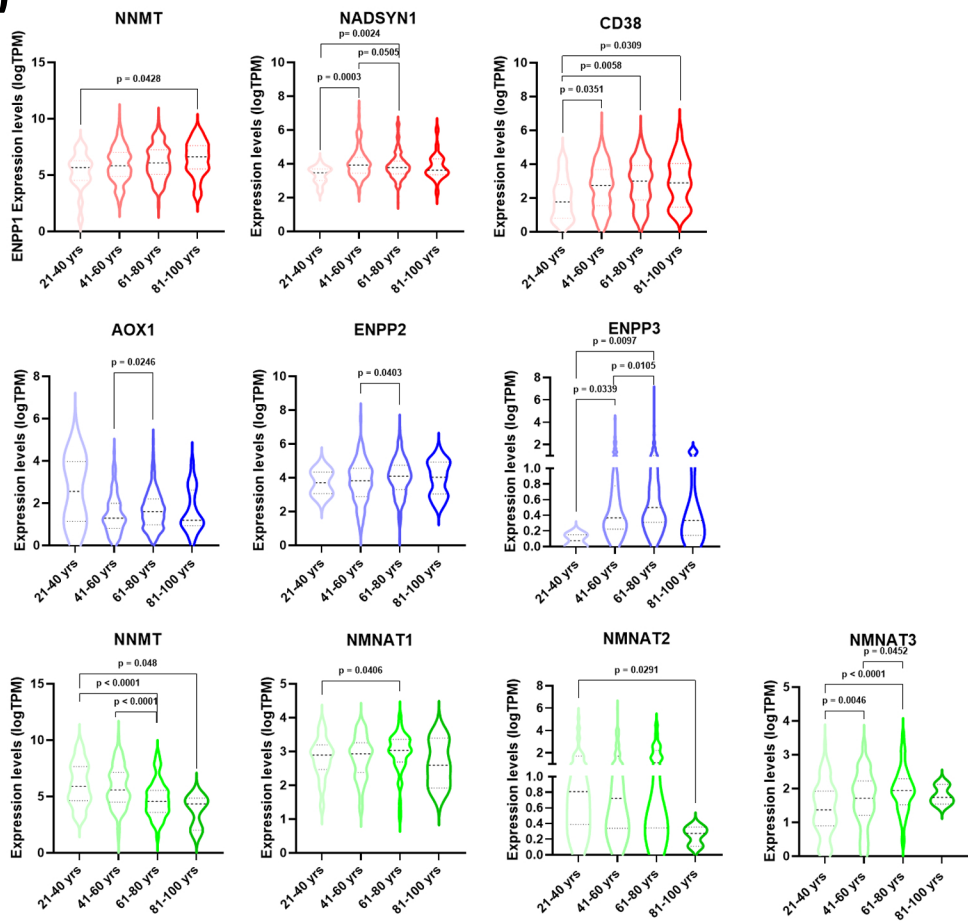**(B)**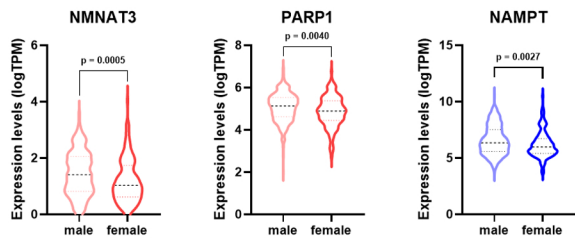

Supplement: Supplementary file 1 [file cancers-16-00309-s001.zip › Figure S1.pdf]

### HNSCC

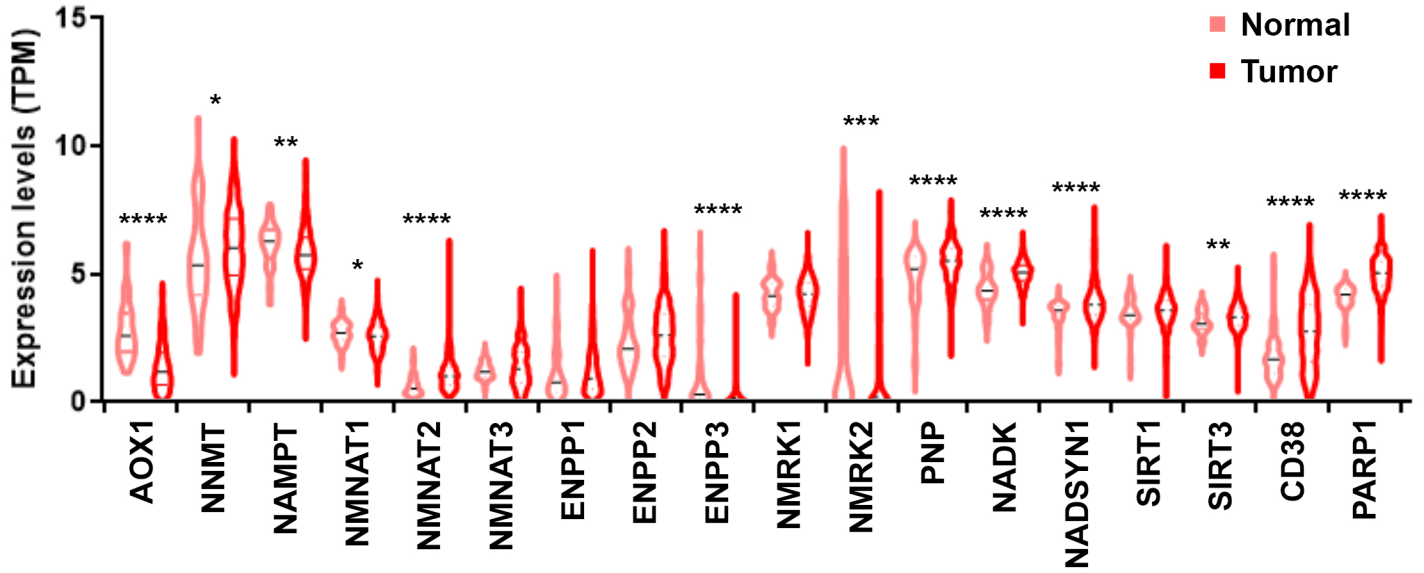

### LuSCC

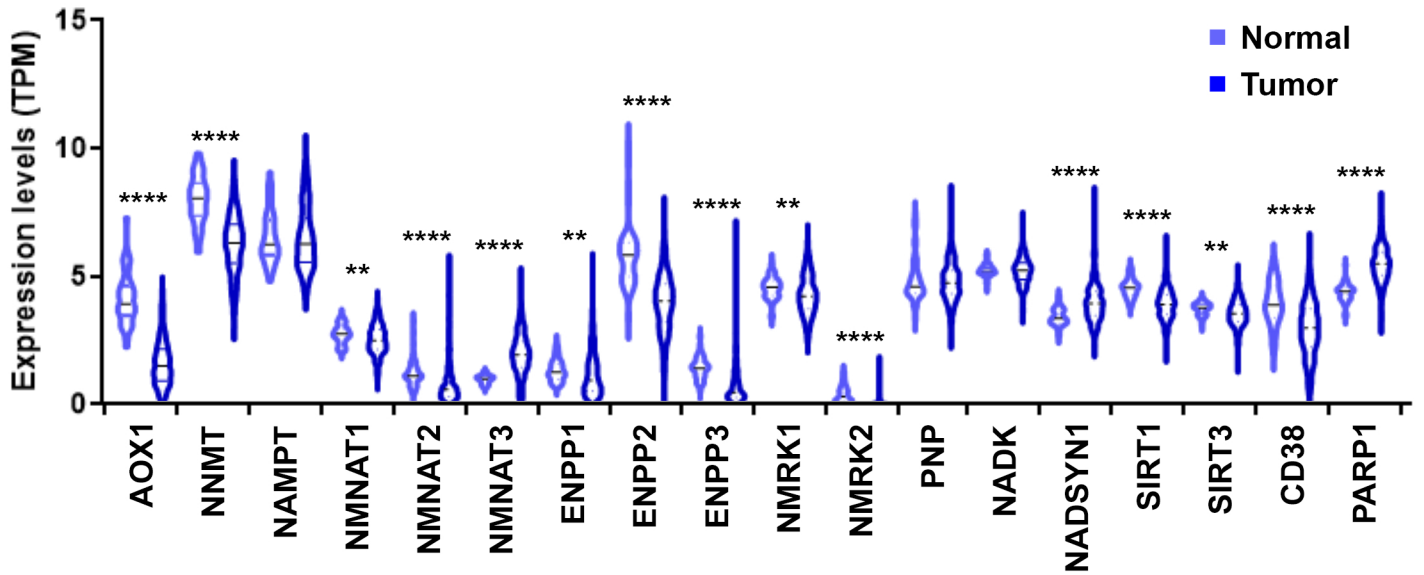

### CeSCC

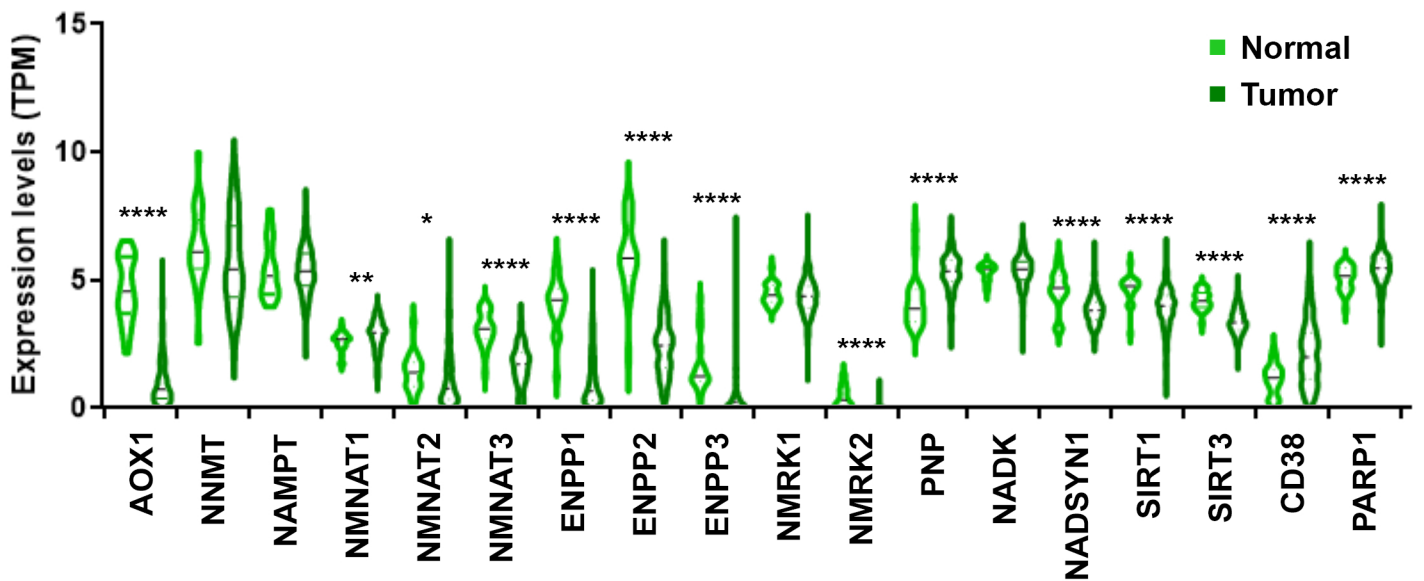

Supplement: Supplementary file 1 [file cancers-16-00309-s001.zip › Figure S2_new.pdf]

# HNSCC

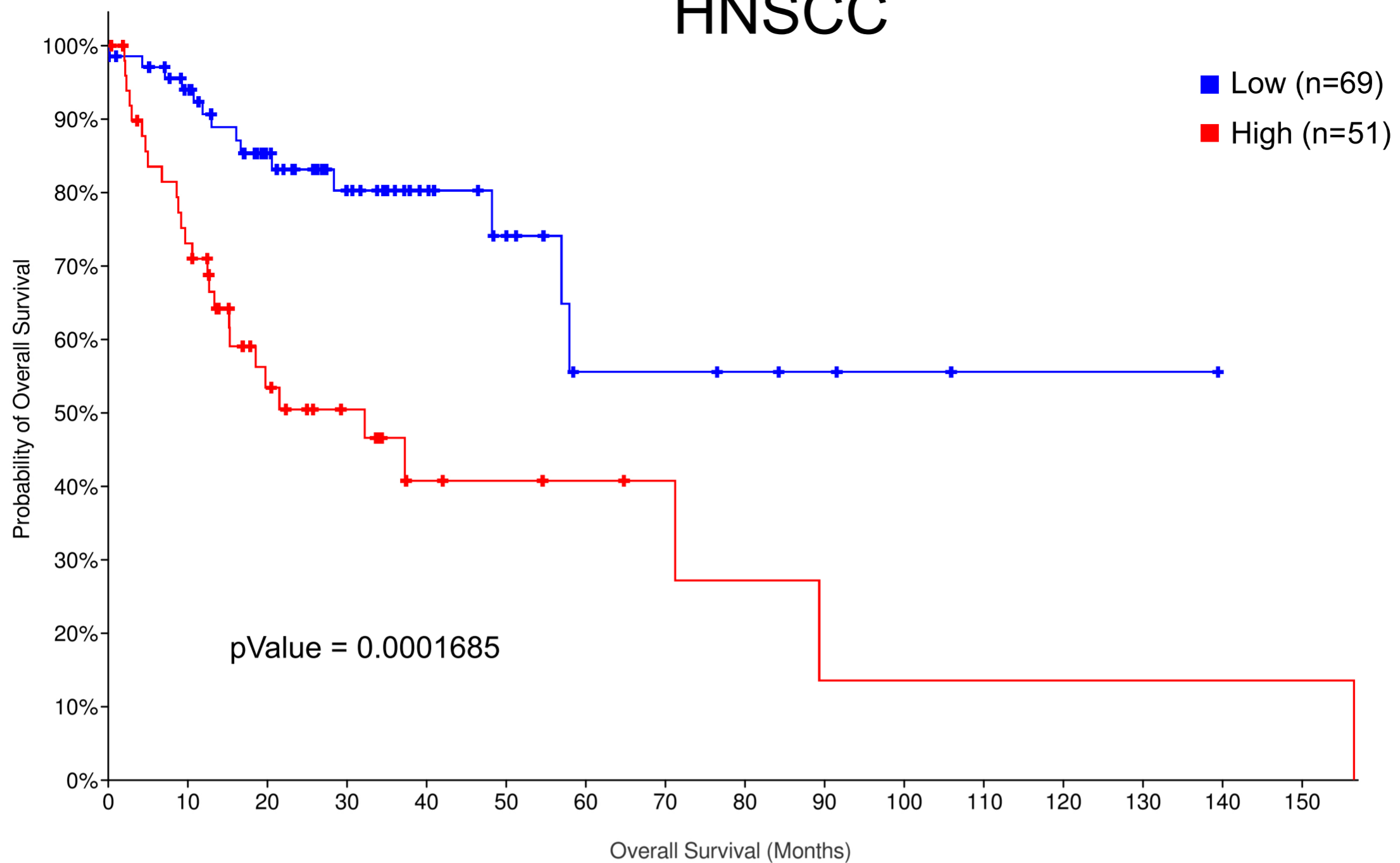

# CeSCC

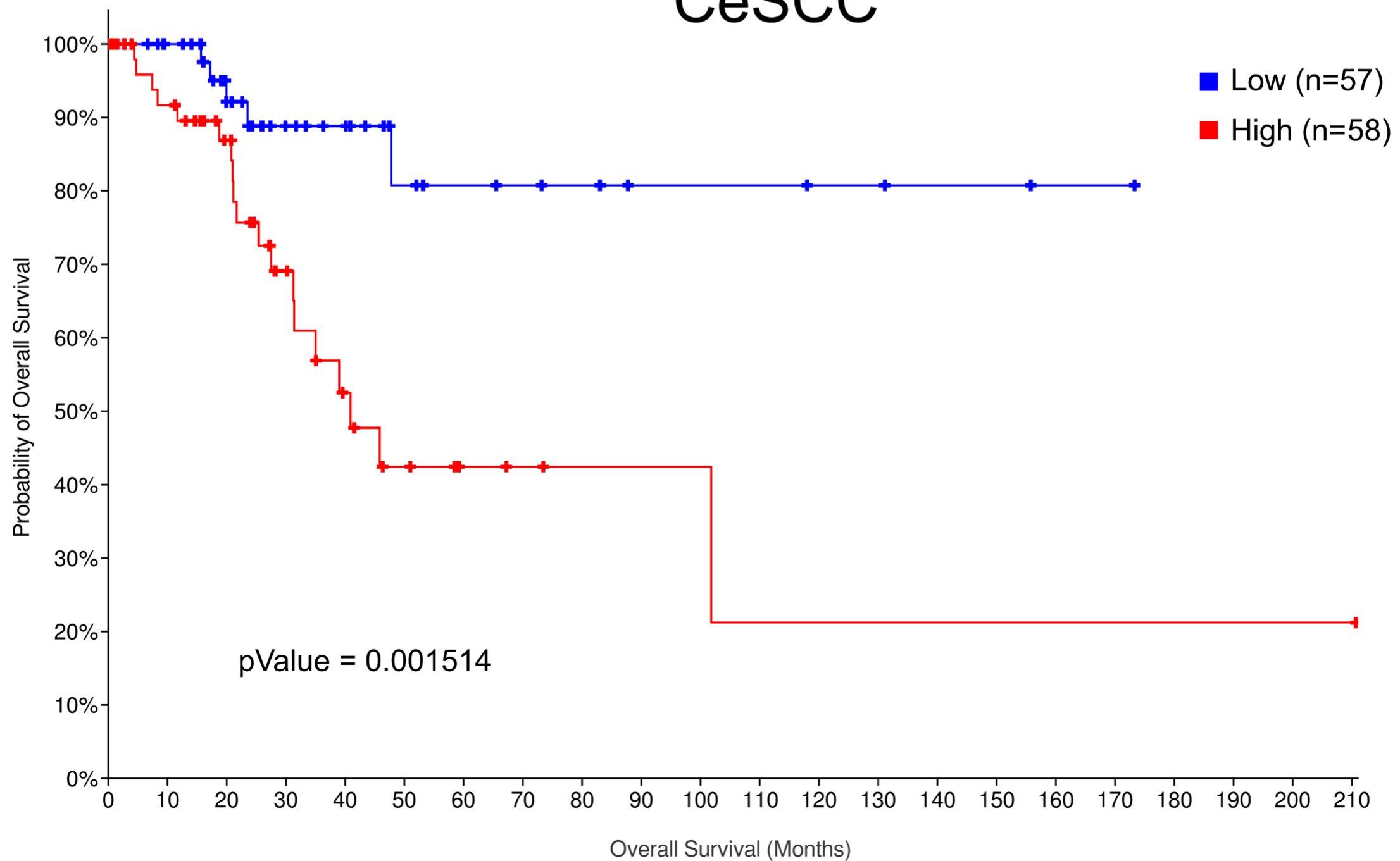

Supplement: Supplementary file 1 [file cancers-16-00309-s001.zip › Figure S4_new.pdf]
